# Vascular Wall-Resident CD44+ Multipotent Stem Cells Give Rise to Pericytes and Smooth Muscle Cells and Contribute to New Vessel Maturation

**DOI:** 10.1371/journal.pone.0020540

**Published:** 2011-05-26

**Authors:** Diana Klein, Philip Weißhardt, Veronika Kleff, Holger Jastrow, Heinz Günther Jakob, Süleyman Ergün

**Affiliations:** 1 Institute of Anatomy, University Hospital Essen, Essen, North Rhine-Westphalia, Germany; 2 Clinic for Thorax and Cardiovascular Surgery, University Hospital Essen, Essen, North Rhine-Westphalia, Germany; University of Bristol, United Kingdom

## Abstract

Here, we identify CD44(+)CD90(+)CD73(+)CD34(−)CD45(−) cells within the adult human arterial adventitia with properties of multipotency which were named vascular wall-resident multipotent stem cells (VW-MPSCs). VW-MPSCs exhibit typical mesenchymal stem cell characteristics including cell surface markers in immunostaining and flow cytometric analyses, and differentiation into adipocytes, chondrocytes and osteocytes under culture conditions. Particularly, TGFß1 stimulation up-regulates smooth muscle cell markers in VW-MPSCs. Using fluorescent cell labelling and co-localisation studies we show that VW-MPSCs differentiate to pericytes/smooth muscle cells which cover the wall of newly formed endothelial capillary-like structures in vitro. Co-implantation of EGFP-labelled VW-MPSCs and human umbilical vein endothelial cells into SCID mice subcutaneously via Matrigel results in new vessels formation which were covered by pericyte- or smooth muscle-like cells generated from implanted VW-MPSCs. Our results suggest that VW-MPSCs are of relevance for vascular morphogenesis, repair and self-renewal of vascular wall cells and for local capacity of neovascularization in disease processes.

## Introduction

New formation of blood vessels has undoubtedly been shown to be essential in physiologic as well as pathologic processes [1], [2]. The vessel wall has usually been thought to be relatively quiescent. While until more than a decade ago it was generally accepted that new blood vessels in the adult are only provided by angiogenesis the discovery of endothelial progenitor cells (EPCs) circulating in the peripheral blood and their contribution to neovascularization led to a crucial revision of this concept [3]. Despite some still controversial findings, today it is widely accepted that new vessels in the adult are formed by angiogenesis and postnatal vasculogenesis [4], [5]. The existence of a vasculogenic zone within the vascular adventitia has recently been identified in adult human vessels. This niche-like zone is believed to act as source of progenitors for postnatal vasculogenesis [6]–[8]. From the literature it is already apparent that a complex interplay between circulating and resident vascular wall progenitors takes place during embryonal and postnatal life. A structural and functional disarray of these intimate stem cell compartments could hamper appropriate vascular repair, the development of vascular disease being the direct clinical consequence in adult life [9].

Beside these progenitors, adult arteries may contain cells with characteristics of ancestral stem cells [7], [10], [11]. Based on these findings someone can hypothesize that a cell type, normally involved in physiological vascular homeostasis, might also act as reservoir of undifferentiated cells ready to supply the cellular demands and acquiring local phenotypic characteristics [12]. Multipotent mesenchymal stem cells (MSCs) would be good candidates for supplying this reserve function. MSCs are multipotent and are commonly characterized by their ability to adhere on plastic, to express a typical panel of surface markers and to differentiate into osteocytes, chondrocytes and adipocytes in vitro. Generally, MSCs are isolated from bone marrow or fatty tissue [13], [14]. There is little information regarding the natural distribution of these cells in different organs and their biology in the living organism. The exact identification of the MSC niche is necessary to validate results obtained in vitro and to further the knowledge of their physiological functions. MSCs are supposed to be one of the most promising types of adult stem cells for cell-based therapies [15]. The establishment of a MSC niche in the vascular adventitia provides a basis for the rational design of additional in vivo therapeutic approaches. Beside bone marrow (BM)-derived MSCs recent studies suggest that the distribution of MSCs throughout the post-natal organism is related to their existence in the vascular adventitia [16], [17]. However, the precise native localization of MSCs and their cellular characteristics in their native niche remains obscure. Furthermore, the precise in vivo MSC attribution remains to be established. Unfortunately, there is no definitive marker allowing the prospective isolation of MSCs from fresh tissue [18], [19]. A recent publication demonstrated that a subtype of CD34+ cells of the vasculogenic zone, which were found to be positive for several MSC markers under certain in vitro culture conditions, possesses the capacity to act as perivascular support cells [20].

Taken together, we hypothesized that the wall of adult blood vessels harbours multipotent stem cells beside the lineage committed progenitors, which may represent an important source for pericytes and smooth muscle cells (SMC) during angiogenesis and postnatal vasculogenesis. Here, we show that CD44(+)CD73(+)CD34(−)CD45(−) VW-MPSCs which exhibit typical MSC characteristics and predominantly reside within the adventitial vasculogenic zone of human arteries. Combining direct labelling with EGFP and immunostaining for specific markers we show that VW-MPSCs isolated from these arteries via CD44+ immunoselection exhibit selective adherence on plastic, differentiate into TAGLN+ cells, and cover the new vessels formed by endothelial cells (EC). Thus, vascular wall-resident MPSCs might serve as a local source for pericytes and SMC in all organs and contribute to stabilization and maturation of new blood vessels; processes of broad spectrum of relevance e.g. in tumor, atherosclerosis, tissue regeneration and therapeutic angiogenesis.

## Materials and Methods

### Reagents and antibodies

Human VEGF165, human PDGF-BB, FGF2 and TGFβ1 were from BioVision (Mountain View, USA). Growth-Factor-Reduced (GFR)-Matrigel was from BD Biosciences (Heidelberg, Germany). TOTO®-3-iodide was from Invitrogen (Karlsruhe, Germany). Mouse anti-human CD44 antibody was from antibodies online (Aachen, Germany), CD90 antibody from ebioscience (San Diego, USA), CD105 and TAGLN antibodies from Acris Antibodies (Herford, Germany), CD105, Stro1, CD73, αSMA, KDR, Oct3/4 and PDGFRβ antibodies were from Santa Cruz (Santa Cruz, USA), rabbit anti-RGS5 antibody was from Invitrogen, calponin1 antibody was from Epitomics (Burlingame, USA), CD34 and CD31 antibodies was from Dako (Hamburg, Germany). cDNA from human ES cells was kindly provided by O. Brüstle (Bonn, Germany). PKH67 Green and PKH26 Red Fluorescent-Cell-Linker Kit for general cell membrane labelling were from Sigma (Steinheim, Germany) and used according to the manufacture's instruction. All peroxidase- and fluorescently-labelled secondary antibodies were from Jackson IR Laboratories (West Grove, USA).

### Tissue and Cells

Human internal thoracic artery (hITA) samples were obtained during surgery (sparse material) according to local ethical and biohazard regulations and provided from the Clinics of Thoracic and Cardiovascular Surgery, University Hospital Essen for our institute. All these studies including human tissue samples were approved by the local ethic committee. Informed consent (written form, Nr.10-4363) was obtained from Ethik-Kommission, University Medical Faculty, Essen, Germany. HAoSMC and HUVEC were from Lonza (Walkersville, USA) and cultivated as recommended by the manufactures in complete SmGM-2 and EGM-2 medium. Human lung adenocarcinoma cell line A549 and prostate carcinoma cell line PC3 was obtained from ATCC (Rockville, USA). Cells were cultured in DMEM (GIBCO, Karlsruhe, Germany) supplemented with 10% FCS, 100 U/ml penicillin and 100 mg/ml streptomycin. For the generation of A549 and PC3 cell-conditioned media cells were incubated in NGM/2% FCS for 24 hours. As control NGM was incubated for the same time at 37°C without cells.

### Isolation and purification of VW-MPSCs

Specimens of hITA were excised under a dissection microscope and contaminating fatty and muscle tissue was removed. After several washes, vessels were mechanically minced and dissociated for 30–40 minutes at 37°C in OptiMEM I medium (GIBCO) containing 0.2% type 2-collagenase (Worthington, Lakewood, USA) and 5 U/ml elastase (Sigma). On dissociation, cells were washed twice in PBS containing 5% FCS (300× g, 10 minutes, 4°C). Cellular suspensions were passed through 70 µm pore size filters. Highly pure VW-MPSCs were generated using a CD44 antibody and MACS technology (Miltenyi Biotec). MACS was used according to the manufacturer's instructions and as described previously [21]. Primary VW-MPSCs were cultivated on plastic cell culture plates using complete human MSC-GM media (PromoCell, Heidelberg, Germany). Medium was removed 24 hours after initial plating, non-adherent cells were washed away and fresh medium was replaced.

### Generation of spheroids

Matrigel plug specimens were performed as previously described [22]. Spheroids containing 100 HUVEC and 100 VW-MPSCs per spheroid were generated using the hanging drop protocol. Indicated cell numbers of HUVEC and MPSCs were mixed in medium containing methocell (20% methocell stock solution and 80% normal growth media) and plated onto non-adherent plastic square petri dishes in 50 µl drops containing 200 HUVEC/MPSCs each. Plates were turned upside down and incubated for 24 hrs in a humidified atmosphere at 37°C. For EGFP labelling VW-MPSC were transfected with the EGFP encoding vector pN1-EGFP using PrimeFect DNA II (Lonza) according to the manufactures instructions and 24 hours prior spheroids generation. Transfection efficiencies were usually about 50%. The next day spheroids were harvested using sterile 5 ml pipettes and 5 ml PBS/10% FCS per dish and collected by brief centrifugation (5 minutes at 150 g, without brake, room temperature). Spheroids were resuspended in pre-cooled GFR-Matrigel (1000 spheroids/300 µl Matrigel) containing the different growth factors (VEGF-A, FGF2: 10 ng/ml each; TGFβ1: 5 ng/ml).

### *In vitro* angiogenesis assay

VW-MPSCs were used to generate spheroids of defined cell number (400 cells/spheroid) and used for in-gel sprouting angiogenesis experiments as previously described [21]. In brief, defined cell numbers of VW-MPSCs were mixed in methocell medium and plated onto non-adherent plastic square petri dishes in 50 µl drops containing 500 MPSCs each. Spheroids were harvested as described above, resuspended in pre-cooled GFR-Matrigel (20–30 spheroids/250 µl Matrigel) and plated in 48-well cell culture plates. After 30 minutes 250 µl NGM was added containing the different growth factors (VEGF-A, PDGF-BB, FGF2: 10 ng/ml each; TGFβ1: 5 ng/ml) or tumor cell conditioned medium (ratio 1∶1). Data are presented as mean ± SD from three (FGF2) and four (VEGF, PDGF, TGF, tumor cell supernatant) independent experiments.

### Animals and Matrigel plug assay

Scid mice were purchased from Janvier (Le Genest-St-Isle, France) and received human care according to the guidelines of the NIH, USA. Animal experiments were approved by the animal ethics committee in NRW, Germany (Regierungspräsidium Düsseldorf Az.8.87-50.10.37.09.183G1050/09). Matrigel plugs were performed and collected as previously described [22]. In brief, 14 mice were anesthetized by injection of intraperitoneal Rompun/Hostaket and the pre-cooled GFR-Matrigel-cell solution (300 µl/injection) was injected subcutaneously. At day 14, mice were killed and plugs were removed. Plug samples were fixed with 4% paraformaldehyde (PFA) and subjected for paraffin embedding and sectioning. Experiments were repeated twice (28 mice in total).

### Trilineage differentiation assay

Differentiation of VW-MPSCs into adipocytes, chondrocytes and osteocytes was done using ready-to-use differentiation media from Lonza (hMSC Differentiation BulletKit – Adipogenic, PT-3004; -Chondrogenic, PT-3003; -Osteogenic, PT-3002) according to the manufactures instructions. Adipogenic differentiation was verified using Oil red staining, chondrogenic differentiation was verified using Collagen type II antibody (Santa Cruz) and immunohistochemitry and osteogenic differentiation was verified using NBT/BCIP staining (Sigma) for alkaline phosphatase activity.

### Tube formation assay (Matrigel Assay)

The tube formation was performed as previously described [21]. In brief, VW-MPSCs were seeded onto GFR-Matrigel in NGM with or without VEGF-A or FGF2 (10 ng/ml). Capillary-like tube formation was analysed using light and confocal microscopy at indicated time points.

### Immunohistochemistry and immunofluorescence

Paraffin embedded tissue sections were hydrated using a descending alcohol series, incubated for 10–20 minutes in target retrieval solution (Dako) and incubated with blocking solution (2% FCS/PBS). After permeabilisation, sections were incubated with primary antibodies over night at 4°C. Antigen was detected with a peroxidase-conjugated secondary antibody (1/250) and DAB staining (Dako). Specimens were analyzed by phase contrast microscopy. For immunofluorescene analysis the antigen was detected with an anti-rabbit Alexa488 and anti-mouse Alexa555-conjugated secondary antibody (1/500). TOTO®-3 iodide was used for nuclei staining. For immunocytochemistry VW-MPSCs were plated on chamber slides (BD Bioscience) prior fixation with 4% PFA, washed (PBS) and incubated with blocking solution (2% NGS/PBS) for 30 minutes. After permeabilisation, cells were incubated with antibodies to mouse anti-CD44, rabbit anti-RGS5, rabbit anti-Sox2 (all 1/100) and to mouse anti-Oct3/4 (1/50). Antigen was detected with fluorescently labelled secondary antibodies as described above. Specimens were analyzed by confocal microscopy.

### RNA isolation and Real-Time RT-PCR (QRT-PCR)

RNA was isolated and QRT-PCR analysis was performed as previously described [21]. Analysis was carried out using the oligonucleotide primers listed in Table S1.

### FACS and Western blot

For FACS analysis 5*10^6^ cells were washed, fixed (4% PFA) on ice (30 minutes), washed and resuspended in 100 µl of 1% BSA/PBS containing diluted antibody (1/100) or corresponding isotype control. After 45 min incubation on ice the cells were washed twice with 1% BSA/PBS and resuspended in 400 µl of the same buffer. For cell permeabilisation 0.5% Saponin was added to the 1% BSA/PBS solution. Stained cells were analyzed with FACS-Calibur™ (BD Biosciences). Obtained results were evaluated with WinMDI software. Whole cell lysates were generated by scraping cells into ice-cold RIPA-P buffer (150 mmol/L NaCl, 1% NP40, 0.5% sodium-desoxycholate, 0.1% sodium-dodecylsulfate, 50 mmol/L Tris/HCL pH 8, 10 mmol/L NaF, 1 mmol/L Na_3_OV_4_, supplemented with complete Protease-Inhibitor-Cocktail (Roche) and performing 2–3 freeze-thaw cycles. Protein samples (50–100 µg total protein) were subjected to SDS-PAGE electrophoresis and Western blots were done as previously described using TAGLN (1/2000), αSMA (1/2500) or βActin (1/5000) antibodies [21].

### Electron microscopy

Matrigel tissues were fixed with phosphate buffered glutaraldehyde (4.5%), contrasted with 1% osmiumtetraoxide and 1% uranylacetate and embedded in EPON®. Thin sections (8 nm) were cut and mounted on 200 Mesh hexagonal cooper grids. For contrast enhancement uranylacetate and leadcitrate were applied. A Zeiss transmission electron microscope (EM 902A) was used for investigation at 80 KV. Digital image acquisition was performed on a MegaViewII slow-scan-CCD camera connected to a PC running ITEM® 5.0 software.

### Statistical Analysis

Paired or unpaired two-tailed t-tests were performed using GraphPad InStat3 software depending on effective matching of analyzed data. SD is indicated by error bars. Significance was assumed for P values <0.05.

## Results

### Localisation of vascular wall-resident putative MSCs

**Figure 1:**
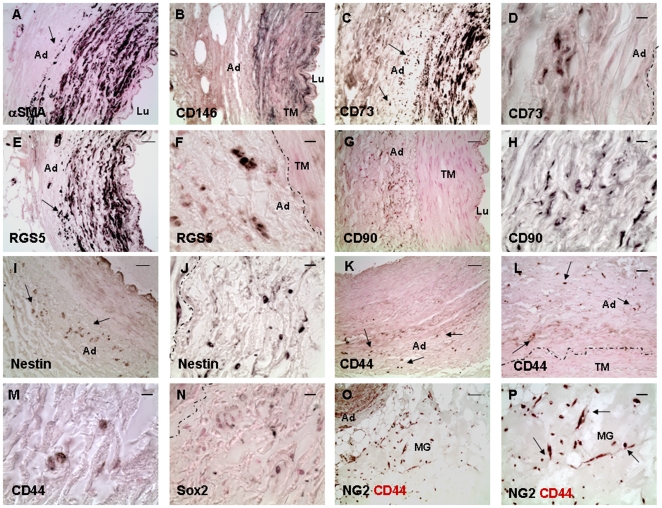
Putative MSCs within the hITA wall. (A) Immunostainings of hITA sections show that αSMA (alpha smooth muscle actin) is mainly detected in SMC, but also in single cells of vasculogenic zone (arrows). (B) CD146 staining is limited to the SMC layer. (C–F) CD73 and RGS5 (regulator of G-protein signaling 5) show the same staining patterns as αSMA. (G–J) CD90 and nestin positive cells are seen in the vasculogenic zone within the adventitia (arrows). (K) CD44 staining is only found in single cells within the adventitia, prominently near to the media (L, M) as visualized by higher magnification (arrows). (N) Sox2 staining is also found in single cells within the adventitia. (O, P) After performing ring assay, when small hITA sections were embedded in GFR-Matrigel and cultured for 2–7 days, after 2–3 days numerous cells positive for both CD44 and NG2 are detectable in Matrigel (arrows) indicating the mobilization and sprouting capacity of the CD44+ putative MSCs from the hITA wall. Lu lumen, TM tunica media, Ad adventitia, MG Matrigel. Dotted line marks the border between media and adventitia of the hITA wall. Bar A–C, E, G, I, K 50 µm; D, F, H, J, M, N 10 µm, L, P 25 µm.

**Figure 2:**
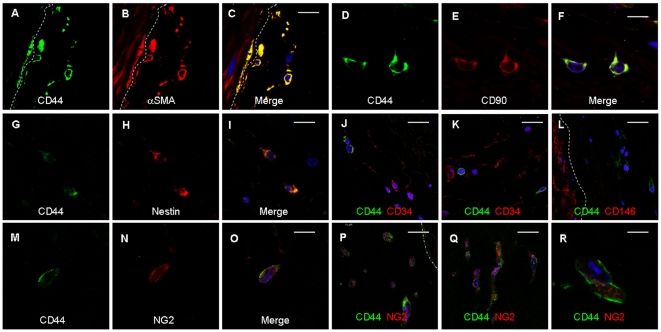
Co-localisation of MSC marker proteins in CD44+ cells within the hITA wall. Double-immunostainings of hITA sections using antibodies against typical MSC maker proteins and CD44 demonstrate that CD44+ cells within vasculogenic zone closed to tunica media (emphasised by dashed line) are also positive for αSMA (A–C), CD90 (D–F), and nestin (G–I), but they are negative for endothelial and hematopoietic progenitor cell marker CD34 (J–K), and lack also CD146 expression (L). Single CD44+ cells within that zone express NG2 (N–O) and these cells migrate into the outside of the vessel wall when performing ring assay studies (P–R; blue, TOTO®-3 iodide).

Conforming our findings published previously [8] we found in arterial ring assay studies αSMA+ cells migrating from the vessel wall and covering new capillaries (Figure S1). Based on such observations we wanted to know which type(s) of vascular wall-resident progenitor or stem cells give(s) rise to these pericytes/SMC. To identify such cells, and to characterize their localisation pattern within the vessel wall precisely and marker proteins that distinguish them from mature SMC we performed immunostaining on sections of adult human internal thoracic artery (hITA). The well known SMC marker αSMA was predominantly found in the SMC layer (tunica media) (Figure 1A) as expected, as well as the markers TAGLN, HAPLN, SM-MHC (not shown). Whereas these markers and CD146 (Figure 1B) were exclusively located within that vessel area, αSMA, and the known MSC marker CD73 and RGS5, a marker for developing pericytes, showed in addition a dot-like distribution within the adventitia, indicating single cells positive for these markers within the vasculogenic zone (Figure 1A,C–F). Remarkably, CD90, nestin, CD44 and Sox2 were present within that zone (Figure 1G–N) clearly indicating the presence of single cells exhibiting MSC characteristics within the vasculogenic zone. Comparable results were obtained from staining of human saphenous vein and radial artery (not shown). In addition, sections were stained with isotype controls serving as controls (not shown). We then performed ring assay studies to test whether CD44+ cells are capable to migrate into the outside of the vessel wall using small hITA fragments. CD44+ cells were found within the Matrigel after 2–3 days of culture (Figure 1O,P) and were also positive for the early pericyte marker NG2. For better characterization of these cells in their native niche we performed double immunostaining on hITA sections using antibodies against typical MSC makers (Figure 2). Single CD44+ cells within vasculogenic zone also express αSMA (Figure 2A–C), CD90 (Figure 2D–F), and nestin (Figure 2G–I), but not CD34 (Figure 2J–K), a marker for endothelial and hematopoietic progenitors and lack also CD146 expression (Figure 2L). Furthermore, single CD44+ cells within that zone express NG2 (Figure 2N–O) and these cells migrate into the outside of the vessel wall when performing ring assay studies (Figure 2P–R). In addition, sections were stained with isotype controls serving as controls (Figure S2).

### Improved purification of vascular wall-derived multipotent stem cells

**Figure 3:**
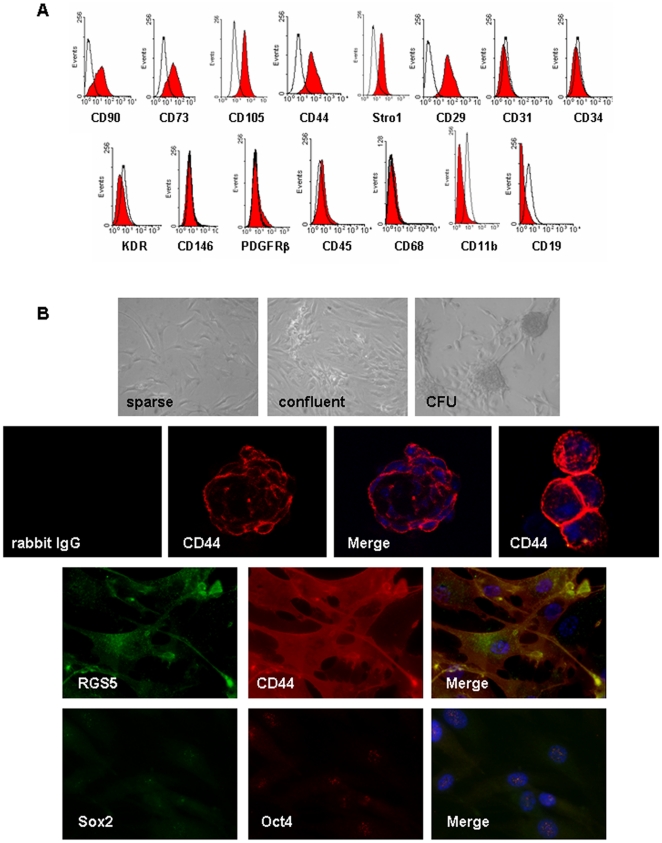
Improved purification and characterisation of vascular wall-derived MPSCs. (A) FACS analysis on MPSCs isolated from hITA wall and selected by MACS using CD44 monoclonal antibody show that they are positive for CD90, CD73, CD105, CD44, Stro1 and CD29 but negative for lineage cell markers CD45, CD68, CD11b, CD19, SMC markers CD146 and PDGFRβ and endothelial cell markers CD34, KDR and CD31 indicating no considerable contamination by other types of progenitors. FACS data representative for at least 3 independent experiments with similar results are shown. (B) Cultivated vascular wall-derived CD44+ cells show flattened and fibroblast-like morphology and form clonally cell aggregates upon prolonged culturing (CFU, colony forming units). Bar 50 µm. Immunofluorescent analysis on cultivated CD44+ MPSCs shows that they are also positive for RGS5 (green) and express stem cell marker Sox2 (green) and Oct4 (red) using confocal microscopy (blue, TOTO®-3 iodide). Bar, 15 µm.

Next, we isolated the CD44+ cells with putative MSC properties from the hITA wall and depleted them of contaminating cell types using MACS-technology in combination with a monoclonal CD44 antibody followed by a selective adherence on plastic dishes in order to characterize them in vitro. The purity of these cell preparations was routinely >95% as analyzed by expression of a marker panel including Stro1, CD105, CD73, CD44, CD90 and CD29 via FACS (Figure 3A), indicating again a MSC-like profile of marker expression. The absence of contaminating cell types such as mature EC or EPCs and HPCs was demonstrated by lack of CD31, CD34, CD45, as well as CD68, CD11b, CD19 expressing cells. Depletion of SMC was shown by the absence of CD146 and PDGFRβ. CD44+ cells plated on plastic dishes showed flattened, fibroblast-like pattern typical for MSCs and they form clonally cell aggregates upon culturing (Figure 3B). Furthermore, isolated CD44+ vascular wall-derived cells were positive for RGS5 as shown by immunofluorescence (IF). Remarkably, they also express the stem cell marker Oct4 and Sox2 at low level (Figure 3C). Sox2 and Oct4 expression was further confirmed on mRNA level using QRT-PCR, as CD44+ cell isolates express quite the same amount of Sox2 as compared to embryonic stem cells and to a lesser extent Oct4 (Figure S3). In order to access clonogenicity and multipotency of isolated CD44+ vascular wall-derived cells, single-cell-derived clones were established by limited dilution in a 96-well plate. In vitro, these cells could be differentiated into adipocytes, chondrocytes as well as into osteocytes (Figure S4) while human aortic SMC (hAoSMC) subjected to the same differentiation procedure did not differentiate into these cell types (not shown). Thus, we will use the term vascular wall-resident multipotent stem cells (VW-MPSCs) in the following for the CD44+ cells isolated from human arterial adventitia.

### In-gel sprouting and proliferation of cultured VW-MPSCs

**Figure 4:**
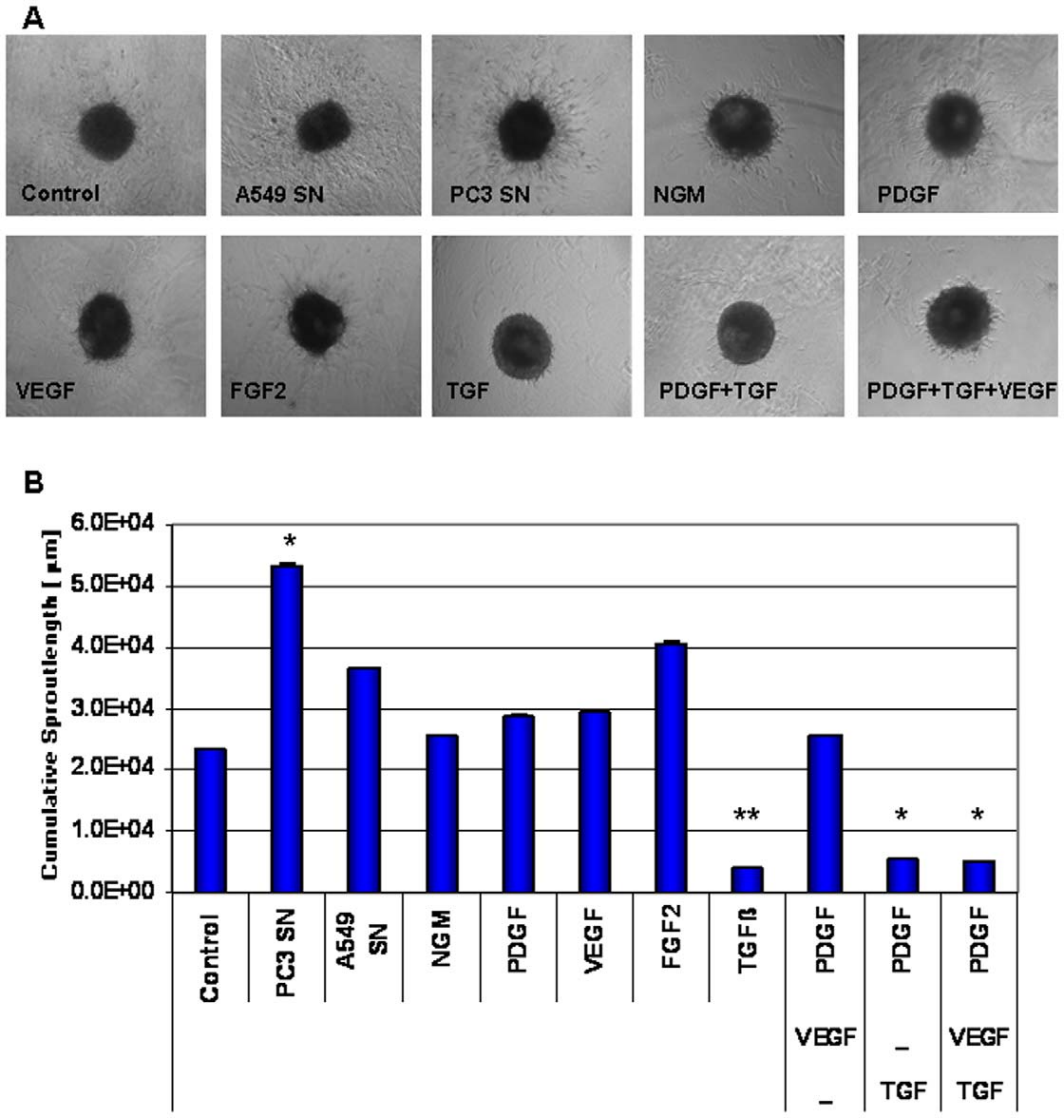
Exogenous TGFβ1 reduces in-gel sprouting of cultured vascular wall-derived MPSCs. Vascular wall-derived MPSCs were embedded in GFR-Matrigel as 3D-spheroids and exposed to normal growth media (NGM), supernatant of the tumor cell lines PC3 and A549 and indicated factors. (A) VW-MPSCs in-gel sprouting and Matrigel invasion was observed by phase contrast microscopy, and (B) quantified after 48 hours of stimulation as shown in the diagram. Application of PC3 and A549 supernatant and FGF2 increases the capacity of cell invasion in the Matrigel while the presence of TGFß1 alone or in combination with indicated factors suppresses it. The data represent the mean cumulative length of all cord-like sprouts growing from 10 individual spheroids per experimental group. The figure shows the results from 1 of 4 independent experiments with similar results. *, p<0.05; **, p≤0.005.

We next investigated the effects of selective growth factors involved in vessel formation and maturation such as VEGF-A, PDGF-BB, FGF-2, TGFβ1 and conditioned media of tumor cells (A549 and PC3) on proliferation and differentiation of VW-MPSCs. We cultured VW-MPSCs as spheroids to test their sprouting capacity into the Matrigel after stimulation with different cytokines (Figure 4A). Despite the high baseline invasion capacity of VW-MPSCs the application of both tumor cell supernatant and FGF-2 additionally increased their invasion capacity (Figure 4B). However, a particularly reduced in-gel sprouting was observed whenever TGFβ1 was present in the medium. The effect of TGFβ1 treatment was reversed when neutralizing TGFβ1 antibody was added in the media (Figure S5A). Next, the number of VW-MPSCs cultured in normal growth media (NGM) with or without application of different growth factors or tumor cell supernatant was quantified after 14 days (Figure S5B). In presence of TGFβ1 alone the total number of VW-MPSC was significantly increased as compared to NGM alone. Increased numbers of VW-MPSCs were found whenever TGFβ1 was present in culture.

### Association and pericyte-like coverage of endothelial tubes by VW-MPSCs

**Figure 5:**
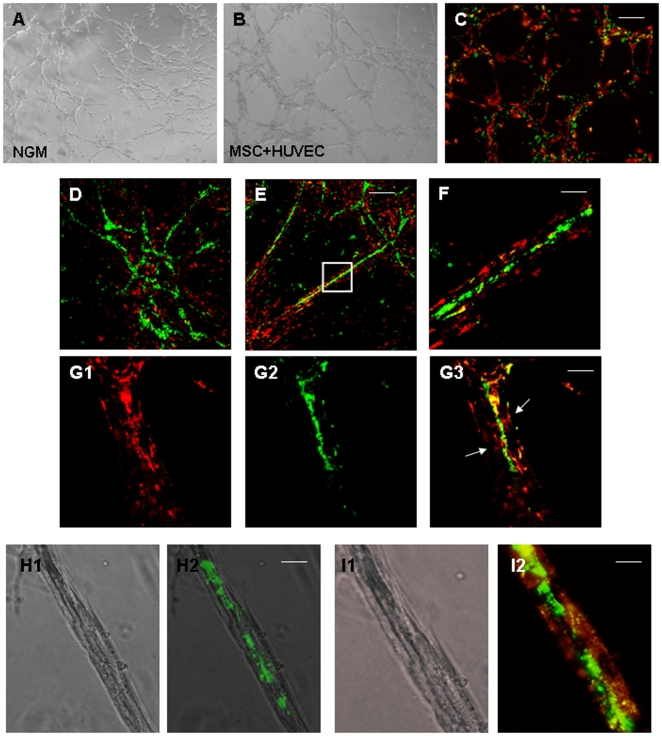
Pericyte-like coverage of endothelial tube-like structures by vascular wall-derived MPSCs *in vitro*. Isolated vascular wall-derived MPSCs (labelled red) are cultured alone or together with HUVEC (human umbilical cord endothelial cells; labelled green) on Matrigel. VW-MPSCs alone form cord-like structures (A) but VW-MPSCs together with HUVEC (B–I) form more prominent network suggesting capillary-like structures in phase contrast microscopy (B). Confocal microscopy analysis after 5–7 hours (C) and after 5 days (D–I) shows a tight association of VW-MPSCs to the capillary-like EC (D). Under additional application of VEGF-A (10 ng/ml) HUVEC form prominent capillary-like structures (green) which are covered by VW-MPSCs (red) (E–I). Higher magnification of these structures reveals a pericyte-like assembly (arrows) of VW-MPSCs to the tube like structures (F–I). Tight association of VW-MPSCs to the capillary-like EC can nicely be seen by combining fluorescence and phase contrast microscopy (H, I). Photographs representative for at least 3 independent experiments with similar results are shown. Bar A–C 50 µm; D,E, 100 µm, F–H15 µm, I 5 µm.

Culturing of fluorescently pre-labelled VW-MPSCs (green) alone or co-culture of them with pre-labelled HUVEC (red) on Growth-Factor-Reduced Matrigel in NGM resulted in cord formation when VW-MPSCs were cultured alone and in capillary-like tube formation when they were co-cultured with HUVEC after 5–7 hours (Figure 5A–C). After 5 days, confocal microscopic evaluation (Figure 5D–I) showed tube-like structures formed by HUVEC through the whole gel and VW-MPSCs were tightly associated with HUVEC. In the presence of VEGF (Figure 5E–I) the tube-like formation by HUVEC and the coverage by VW-MPSCs were more pronounced as compared to NGM alone. Higher magnification revealed that tightly associated VW-MPSCs are wrapped around the capillary-like structures (Figure 5G–I). Similar results were obtained by FGF2 and TGFβ1 application (not shown). The pericyte-like behaviour was also observed when the fluorescently pre-labelled VW-MPSCs were co-cultured with HUVEC as spheroids in Matrigel (Figure S6).

### Differential expression of VW-MPSCs vs. SMC marker genes and VW-MPSCs differentiation into SMC

**Figure 6:**
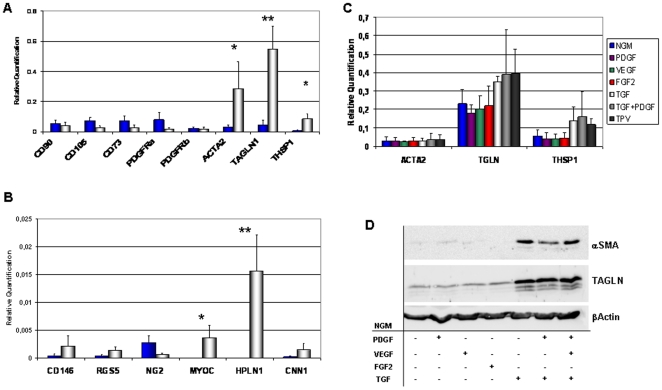
Differential expressions of marker genes in vascular wall-derived MPSCs versus SMC. (A) QRT-PCR analyses show that genes specific for SMC such as alpha smooth muscle actin (αSMA, ACTA2), TAGLN1 (transgelin), THSP1 (Thrombospondin 1), MYOC (myocardin) and HPLN1 hyaluronan and proteoglycan link protein 1 are expressed significantly higher in hAoSMC (human aortic smooth muscle cells) in comparison to MSCs while PDGFRα (platelet-derived growth factor α) and NG2 are expressed stronger in vascular wall-derived MPSCs (A–B). Stimulation of VW-MPSCs with VEGF165, PDGF-BB, FGF2 (10 ng/ml), TGFβ1 (5 ng/ml) alone or in indicated combinations for 14 days shows an up-regulation of SMC markers TAGLN and THSP1 as compared to VW-MPSCs cultured in normal growth media (NGM) (C). Resulting expression levels were normalized by division through the mean expression value of the reference gene (β-actin). Data are presented as mean ± SD from four independent experiments measured at least two times each. *, p<0.05; **, p≤0.005. The stimulation of VW-MPSCs by TGFß1 alone or in combination VEGF and PDGF also increases the protein level of SMC markers αSMA and TAGLN as shown by immunoblotting (D). Total cell lysates were generated by scraping cells in ice-cold RIPA buffer. Equal protein amounts were subjected for SDS-PAGE. TAGLN was detected by Western blot using chemiluminescence. β-actin was included as a loading control. Data representative for at least 3 independent experiments with similar results are shown.

In order to identify unique and moreover suitable genes selectively expressed in VW-MPSCs versus mature SMC, QRT-PCR analyses were performed (Figure 6A–B). The known MSC markers CD90, CD73 and CD105 were found to be slightly higher expressed in VW-MPSCs whereas PDGFRα and NG2 were clearly stronger expressed. Comparable expression levels were detected for PDGFRβ. The known SMC markers ACTA2, TAGLN, HAPLN, CNN1, CD146, RGS5, thrombospondin 1 (THSP1), MYH11 and desmin (not shown) were found to be stronger expressed in SMC as compared to VW-MPSCs. We then investigated the expression of SMC markers in our VW-MPSCs under different culture conditions in order to analyse their putative differentiation into SMC. Therefore, VW-MPSCs were incubated in NGM supplemented with indicated alone or in combination for 14 days. Total RNA of these VW-MPSCs was used to determine the expression levels of αSMA/ACTA2, TAGLN and THSP1 by QRT-PCR (Figure 6C). Exogenous TGFβ1 treatment resulted in an increased expression of the SMC genes TAGLN, THSP1 and HAPLN (not shown) in cultured VW-MPSCs. Western blot analysis confirmed an increase of TAGLN upon TGFβ1 treatment and also of αSMA (Figure 6D). When VW-MPSCs were cultured together with HUVEC und subjected for the same treatment an even higher expression of TAGLN was observed (Figure S7).

### Contribution of VW-MPSCs to new vessel morphogenesis *in vivo*

**Figure 7:**
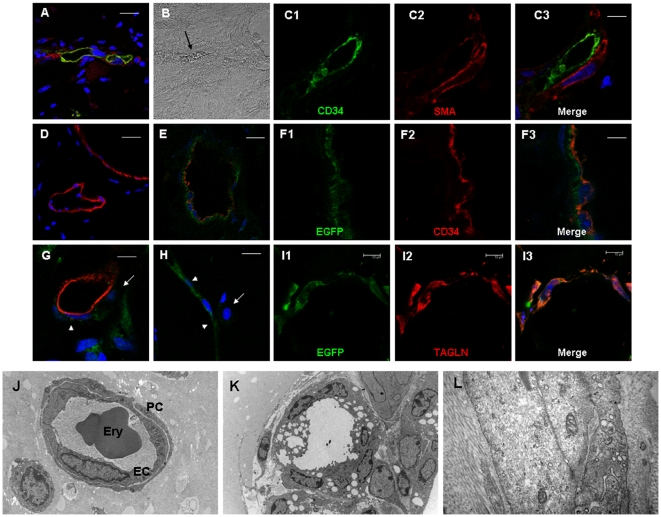
Contribution of vascular wall-derived MPSCs to new vessel formation *in vivo*. VW-MPSCs and HUVEC were grafted into Scid mice subcutaneously for 14 days as spheroids in Matrigel supplemented with growth factors. Immunofluorescent analysis of isolated plug tissues was performed. Double-staining for hCD34 (green) and αSMA (red) shows a close assembly of αSMA+ cells to the vessel wall formed by HUVEC (A, C).Functionally perfused blood vessels within the plugs are identified by presence of erythrocytes within the vessel lumen as detected by phase contrast microscopy (B, arrow). The specificity of hCD34 was demonstrated by its absence in blood vessels of normal mouse fatty tissue (D). Furthermore, double stainings for hCD34 (red) and TAGLN (green) (E–I) show flattened TAGLN+ positive cells in tight association to vessels formed by implanted HUVEC within the plugs (E–G). Intensive vascularisation of plugs is also seen when VW-MPSCs/HUVEC are grafted in Matrigel together with VEGF and FGF2 (A–F, I) as well as TGFβ1 alone (G, H). Cells strongly positive for TAGLN surround tightly the vessels formed by HUVEC (G, arrowheads) while also some single roundly shaped and TAGLN negative cells (arrow) are present (G, arrow). Few flattened and TAGLN+ cells (arrowhead) which are not assembled to new vessels are found in Matrigel indicating the presence of SMC, while some rounded cells (arrowheads) are only weak positive for TAGLN which probably represent still differentiating MPSCs (H). EGFP-labeling of VW-MPSCs shows co-localization of TAGLN (red) and EGFP fluorescence identifying the EGFP-labeled VW-MPSCs as the source of the pericytes and SMC-like cells surrounding the vessels (blue, TOTO®-3 iodide) (I). Bar A, C 20 µm; E, H, I 10 µm; B, F, G 5 µm. Electron microscopic analysis demonstrates a capillary with endothelial cells (EC) and regularly assembled pericytes (PC) covering endothelial cells. The presence of erythrocytes (Ery) within the capillary lumen indicates the connection of this capillary to the blood perfusion (J). In some areas immature vessels are seen as observed by EC morphology and absence of pericytes in the vessel wall (K). Also single cells or small cell clusters with contractile filaments in the cytoplasm are found indicating the presence of SMC, probably generated from the implanted SMCs as shown by higher magnification (L).

Finally, we studied the contribution of VW-MPSCs to morphogenesis of functioning blood vessels *in vivo* using a co-xenotransplantation of VW-MPSCs/HUVEC as spheroids in Matrigel which were implanted subcutaneously into Scid mice with addition of selective angiogenic cytokines (Figure 7). After 14 days Matrigel plugs were isolated and subjected for immunohistochemistry for human-selective CD34 (hCD34), αSMA and TAGLN. Within the plugs the formation of new blood vessels was demonstrated by phase contrast microscopy and by vessels lined by HUVEC as shown by hCD34 staining (Figure 7A–C). Co-staining for αSMA shows that αSMA+ cells are closely associated to these vessels and they achieve a more flattened and elongated phenotype indicating the potential differentiation of co-implanted VW-MPSCs into SMC. The results were confirmed by double staining for hCD34 and TAGLN (Figure 7G,H) showing a close association of TAGLN+ cells to the new vessels visualized by hCD34+ HUVEC. Strongest vascularization of plug tissue was observed when VW-MPSCs and HUVEC together were grafted in Matrigel and stimulated by combined application of VEGF and FGF-2 or TGFβ1 alone (Figure S9C). Beside flattened TAGLN+ cells with long processes which are tightly integrated into the vessel wall as pericytes/SMC we found rounded cells with big and rounded nuclei in close vicinity to the vessels formed by HUVEC which were either negative or stained only weakly for TAGLN (Figure 7G, S8A). They probably represent less or non-differentiated VW-MPSCs. Furthermore, even within plug areas where VW-MPSCs were not directly associated to HUVEC a strong TAGLN staining could be detected in flattened and elongated cells indicating their potential differentiation into SMC whereas undifferentiated or less differentiated VW-MPSCs exhibited weak TAGLN staining (Figure 7H). TAGLN-immunoreactivity was used in order to quantify the extent of pericytes and SMC differentiation (Figure S9). When VW-MPSCs were grafted together with HUVEC and stimulated by combined application of VEGF and FGF-2 71%±19 of all the cells within the plug differentiated into pericytes/SMC, respectively and 70%±16 when TGFß was used. When VW-MPSCs were grafted alone and stimulated with TGFß 63%±16 of all the cells were differentiated into pericytes/SMC. Only a low amount of differentiation (15%±9) was observed within the plugs when VW-MPSCs were implanted without the addition of growth factors. Since no antibody was available recognizing specifically the human SMC or VW-MPSCs we used direct fluorescent labeling of VW-MPSCs via transfection for EGFP. EGFP-labeled VW-MPSCs were then implanted into SCID mice together with HUVEC as described above in order to directly follow their differentiation into pericytes/SMC and integration into the vessel wall. These analyses showed a tightly assembling of EGFP-positive VW-MPSCs to new vessels within Matrigel in pericyte-like manner (Figure 7E,F,I). TAGLN immunostaining demonstrated the co-localization of TAGLN and EGFP fluorescence identifying the EGFP labeled VW-MPSCs as the source of the pericyte/SMC-like cells surrounding the vessels (Figure 7I, S8C). In addition, staining for the pericytes and SMC makers RGS5, αSMA, and CD146 and co-localisation of EGFP-fluorescence confirmed that the EGFP-labeled VW-MPSCs are the source of the pericytes and SMC-like cells surrounding the new vessels (Figure S8C–F). Finally, electron microscopic analyses of plugs showed capillaries with regular assembly of pericytes (Figure 7J) which apparently are connected to the blood perfusion as recognizable from presence of erythrocytes in plug vessels. Early immature vessels formed only by EC (Figure 7K) as identified by presence of Weibel-Pallade bodies were found frequently accompanied by single cell groups with contractile filaments in the cytoplasm indicating SMC as shown by higher magnification (Figure 7L). Furthermore, still roundly shaped cells, probably corresponding to undifferentiated or less differentiated VW-MPSCs were found within the plug.

## Discussion

Here, we show for the first time that CD44+ VW-MPSCs exhibiting major characteristics of MSCs predominantly reside in the so-called vasculogenic zone of vascular adventitia and give rise to generation of pericytes/SMC which in turn are assembled to the wall of new vessels. Studies performed during the last five years showed that not only embryonic and fetal aortas but also adult human blood vessels harbour EPCs and hematopoietic stem cells (HPCs) in their walls [6], [8], [23], [24]. Supporting these findings, recently the presence of Sca1+ cells in murine vascular adventitia was shown which differentiated into SMC in vitro [25]. More recently, it was shown that a subset of CD34+ vascular wall-resident progenitors with clonogenic and proangiogenic potential act as paracrine stimulants for vascular SMC [20]. Within that publication Campagnolo et al. have shown that a subtype of CD34+ cells from the vasculogenic zone of human saphena veins became negative for CD34 under certain in vitro culture conditions and were found to be positive for several MSC markers (saphena vein-derived progenitor cells, SVPs). In a model of ischemia these cells were shown to act as perivascular support cells, most likely to be pericytes [20]. However, the author did not perform co-localisation studies for SVP and SMC marker proteins after transplantation, which were necessary in order to clearly demonstrate the pericyte/SMC differentiation of those cells. We hypothesized the existence of mesenchymal vascular wall-resident stem cells with the capacity to differentiate into pericytes/SMC. Here we use a single marker, CD44 to isolate this VW-MPSC. These cells lack CD34 expression in situ as well as upon culturing. We suggest that our CD44+ VW-MPSCs represent a different type of multipotent adventitial cells of adult vessels wall as compared to CD34+ SVP cells. Finally, it cannot be totally excluded that CD44+ VW-MPSCs can achieve characteristics of CD34+ SVP upon differentiation processes in situ or in vitro under certain circumstances not studied here.

The vascular adventitia acts as biological processing centre for release of key regulators of vessel wall function and in response to stress, atherosclerotic plaques [6], [10] or injury resident adventitial stem and progenitor cells [23] can be activated and specified to exhibit different functional and structural behaviours [24], [26]. We used several markers in our analyses. Only CD44 resulted in identifying of cells which were present exclusively within the vasculogenic zone. CD44+ cells isolated from freshly prepared hITA fragments exhibit a profile of cell surface markers which is characteristic for MSCs. Since these cells did not express CD146 and PDGFRβ they could be distinguished from vascular SMC. Together with their capability to adhere on plastic and to differentiate along the mesodermal lineage vascular wall-resident CD44+ cells exhibit properties of multipotent stem cells, as they fulfil all the criteria defined by The International Society for Cellular Therapy [27]. Thus, they were named as vascular wall-resident multipotent stem cells.

Already in 1998 Andreeva et al. [28] demonstrated the presence of cells expressing the pericyte marker 3G5 in the subendothelial space and the outer layer of the tunica media. Furthermore, they found a continuous subendothelial network of pericyte-like cells in human vascular bed. Consistent with these findings multipotent MSCs from subendothelial space of saphenous veins were isolated that differentiated into osteoblasts, chondrocytes and adipocytes in vitro [29]. We already postulated the potential presence of MSCs within the adventitial vasculogenic zone as we identified this zone as a niche for EPCs and HPCs [8]. Supporting this hypothesis, in recent report angiogenic mesenchymal stromal cells were obtained from thoracic aortas of multiorgan donors with the ability to differentiate into EC in vitro [11]. Furthermore, it was hypothesized that MSCs were situated throughout the body as pericytes. They are in physical contacts with EC via gap junctions and express at least one marker attributed to pericytes but not pan-endothelial markers [19]. Once these cells are liberated from the endothelial layer the authors suggest to reconsider them as MSCs. Based on our data we suggest reconsidering the “MSCs” from the subendothelial space to be pericytes as they fulfil all the criteria, especially due to their close contact to EC and basal lamina of the capillary wall. Conclusively VW-MPSCs derived from the vasculogenic zone fulfil all criteria of MSCs and in contrast to the pericytes they do not need a retransformation process to achieve a stem cell-like behaviour. Herein, lack of CD146 expression in our VW-MPSC might represent a special feature of VW-MPSCs derived from the vasculogenic zone as compared to pericytes. Studying the well known MSC marker and their expression profiles in VW-MPSCs as compared to SMC we show here that expression patterns highly overlap and this might indicate that there is an urgent need in identifying additional cells type specific markers. Moreover, using hITA tissue sections and performing arterial ring assays we show here that CD44+ VW-MPSCs are mobilised from the adventitia, migrate outside the vessel wall and cover the endothelial capillary-like outgrowth.

Several groups have suggested the use of MSCs derived from BM, adipose tissue and embryonic stem cells to generate SMC [30]–[32]. Increasing awareness points out that association of EC with pericytes/SMC is critical for the proper vascular development, stabilization and maintenance [33]–[35]. Several studies have suggested that pericytes and SMC may play a central role in tumor angiogenesis and determine the success of anti-angiogenic therapies. Thus, a great interest in identifying pericytes in tumor tissue specimens has developed [36]. On the other hand vascular stabilization is crucial for new vessels to survive and to achieve functional properties needed for an adequate perfusion by blood according to the tissue demands. Our data show that CD44+ VW-MPSCs are capable to differentiate into TAGLN+ cells which contain contractile filaments in electron microscopic analyses and cover the in vitro formed endothelial tube-like structures in a pericyte-like manner. While pericytes have mostly been attributed to be involved in relatively late events during neovascularisation such as vessel stabilization, permeability barrier formation and blood flow regulation, pericytes also have been shown to be present during the initial stages of microvessel formation and may even be involved in initiating of microvascular development [37]–[39]. Accordingly, the relationship between pericytes and EC determines the entire life of microvessels. In line with this hypothesis the early presence of pericytes in vascularising tissues and the ability of these cells to form pericyte networks in the absence of EC have been reported [40].

VW-MPSCs contribute to in vivo vessel morphogenesis as we show here by co-implantation of VW-MPSCs and HUVEC in Matrigel plug assay. Within the plugs implanted HUVEC formed blood perfused vessels as shown by presence of erythrocytes within the vessel lumen and hCD34-staining. Co-implanted VW-MPSCs were differentiated into SMC/pericytes and assembled then to the new vessels as shown here by using EGFP-labeled VW-MPSCs. Immunofluorescence analysis of different SMC/pericyte markers undoubtedly confirmed that CD44+ VW-MPSCs have the capability to differentiate into pericyte/SMC and contribute to morphogenesis of new vessels under in vivo conditions. Finally, our electron microscopic studies show at the ultra structural level that these cells are not only assembled to new capillaries but they are regularly integrated into the wall of new capillaries e.g. EC and pericytes are enclosed by the same basal lamina. Moreover, our present findings show that isolated CD44+ MPSCs exposed to exogenous TGFβ1 during culturing exhibit alteration in gene expressing profile by significantly increased expression of the SMC markers TAGLN, HAPLN and THSP1. These factors have been shown to play an essential role in differentiation and proliferation of SMCs [32], [41], vascular morphogenesis [42], and maintenance [41], [42]. TAGLN is expressed exclusively in smooth muscle-containing tissues of adult mammals and is one of the earliest markers of differentiating SMC. However, the reliance on αSMA (ACTA2) expression as a sole criterion for differentiation of a stem or progenitor cell into SMCs is critical and can lead to the false conclusion that the stem cell type being studied has the capacity to produce functional SMCs. Accordingly, our microarray studies revealed that several genes are differentially expressed in vascular wall-derived MSCs in comparison to SMC and TAGLN, ACTA2, THSP1, HAPLN and MYOG are some of the most up-regulated genes in SMCs (unpublished data). Combining the expression pattern of well known MSCs and SMC markers, we were able to analyze the effect of different growth factors on VW-MPSC differentiation into SMC. The increased protein levels of TAGLN and αSMA of cultivated VW-MPSCs stimulated with TGFß1 demonstrate that VW-MPSCs achieve SMC-like pattern. Further studies will be needed in order to better characterize the pathway of TGFb1-induced SMC differentiation of VW-MPSCs.

### Conclusion

Taken together, our results demonstrate that human vessels harbour not only EPCs but also other types of stem cells as shown here for VW-MPSCs which are capable to differentiate into SMC. Our extensive morphogenetic studies and functional analyses identify vascular MPSCs residing predominantly in the vasculogenic zone of adult human blood vessels and provide new mechanistic insights into their potential to differentiate into SMC and assemble to the wall of new vessels. Localized within the vascular adventitia which serves as an interface between the inner parts of vessel wall inclusively blood flow and the surrounding tissue the VW-MPSCs might serve an important therapeutic target. On the other hand, the therapeutic potential of these cells in ischemic disorders and tissue vascularisation as well as in tissue engineering and regeneration is self-evident but needs further studies. Hypothetically, VW-MPSCs can be mobilized from adventitia to the media and differentiate to SMC in cases of injury or damage of the arterial wall cells in order to replace them. Also the cells might contribute to vessel remodeling during embryonic and fetal development. Finally, it is conceivable that the adventitial CD44+ cells contribute to the stabilization of vasa vasorum, an important system of the wall of large and middle sized arteries for the own blood supply.

## Supporting Information

Figure S1 **Arterial sprouting assay in Matrigel using fragments of hITA.** The capillary-like sprouts from aortic wall demonstrate cellular components (arrows) tightly associated to the vessel sprout from the outside (A). Immunohistochemistry for αSMA on sections from such sprouting tissue shows αSMA-positive (arrows) cells covering the capillary-like structure (red staining) visualized by counterstaining via Calcium red (B).
(TIF)

Figure S2 **Control stainings of hITA sections for immunofluorescent analysis.** In immunofluorescent analysis for CD44+ cells in their native niche we performed double immunostainings on hITA sections combining antibodies against RGS5 (rabbit IgG) and mouse isotype control (A), against CD44 (mouse IgG) and rabbit isotype control (B), as well as mouse and rabbit isotype controls (blue, TOTO®-3 iodide). Dotted line marks the border between media and adventitia of the hITA wall.
(TIF)

Figure S3 **Expression of pluripotent stem cell marker genes in vascular wall-derived MPSCs vs. embryonic stem (ES) cells.** QRT-PCR analyses show that genes specific for pluripotent ES (blue bars) cells are partially expressed in VW-MPSCs (grey bars) (Sox2 and to a lower extent Oct4). Y-axis is presented in logarithmic scale. Data are presented as mean ± SD from three independent experiments measured at least two times each.
(TIF)

Figure S4 ***In vitro*** **differentiation of cultured vascular wall-derived CD44+ MPSC.** (A) Cultivated vascular wall-derived CD44+ MPSCs differentiate into adipocytes (Oil red), chondrocytes Coll II) and into osteocytes (ALP) within 14 days after induction of differentiation as shown by Oil red staining, by immunostaining for collagen type II (Coll II) and by histochemical staining for alkaline phosphatase (ALP) as well as the von Kossa staining to visualise mineralised calcium (upper figure panel). No specific staining is seen in the corresponding controls (lower figure panel). Magnification ×20. (B) Cultured CD44+ MPSC were clonally expanded by plating primary cell isolates in 96-well cell culture plastic dishes (1 cell per well) as previously described by Chen et al. [R1]. Developed clones were subsequently subjected to obtain a single cell suspension by accutase treatment and re-plated in 96-well plastic dishes (1 cell per well) in order to generate subclones (sc). Different sub-clones (differentiation I, II) differentiate into adipocytes (Oil red staining), into osteocytes (ALP staining) and into chondrocytes (Coll II staining) within 14 days after induction of differentiation. Magnification ×20. [R1] Chen FG, Zhang WJ, Bi D, Liu W, Wei X, Chen FF, Zhu L, Cui L, Cao Y. Clonal analysis of nestin(−) vimentin(+) multipotent fibroblasts isolated from human dermis. J Cell Sci. 2007 Aug 15;120(Pt 16):2875–83.
(TIF)

Figure S5 **Exogenous TGFβ1 reduces in-gel sprouting and increases cell number of cultured vascular wall-derived MPSCs.** (A) VW-derived MPSCs alone (blue bars) or together with endothelial cells (purple bars; ratio 1∶1) were embedded in GFR-Matrigel as 3D-spheroids and exposed to NGM, TGFβ1 (5 ng/ml) or TGFβ1 and TGFβ1 neutralizing antibody (2 µg/ml). In-gel sprouting was quantified after 48 hours of stimulation. The data represent the mean cumulative length of all cord-like sprouts growing from 10 individual spheroids per experimental group. The figure shows the results of 1 of 2 independent experiments with similar results. (B) VW-MPSCs cultured in NGM supplemented with VEGF165, PDGF-BB, FGF2 (10 ng/ml) and TGFβ1 (5 ng/ml) as well as in control and conditioned media of tumor cell lines A549 (A549-SN) or PC3 (PC3-SN) for 14 days show increased cell numbers in response to TGFß alone or in the indicated combinations. Data are presented as mean ± SD from 3 independent experiments performed in duplicates each. ** p≤0.005.
(TIF)

Figure S6 **Pericyte-like coverage of endothelial tubes by vascular wall-derived MPSCs in an angiogenic sprouting assay.** Pre-labelled VW-MPSCs (green) were seeded together with pre-labelled HUVEC as spheroids (red; ratio 1∶1) in GFR-Matrigel. Capillary-like tube formation was observed within MPSCs/HUVEC after 48 h of culturing. Confocal microscopic analysis was done (A, B1). Bar A3 100 µm, A4 20 µm. Vascular wall-derived MPSCs tightly associate to the tubes formed by HUVEC and wrap them in a pericyte-like manner after prolonged co-culturing (B). Bar 10 µm. Cross-sectioning of samples was followed by a HE-staining in order to demonstrate lumen formation within the capillary tube formation (C, D). Bar10 µm.
(TIF)

Figure S7 **QRT-PCR analyses of vascular wall-derived MPSCs differentiation into SMC upon co-culturing with HUVEC.** VW-MPSCs were cultured together with HUVEC (ratio 1∶1) in normal growth media (NGM) or supplemented with VEGF165, PDGF-BB, FGF2 (10 ng/ml), TGFβ1 (5 ng/ml) alone or in indicated combinations. After 14 days cells were trypsinised and subjected for MACS sorting. HUVEC cells were depleted via immunomagnetic beads using CD34 and CD31 antibodies. Total RNA was harvested from VW-MPSCs extracts and subjected for QRT-PCR analysis of TAGLN, HAPLN and CNN1 expression. Resulting expression levels were normalized by division through the mean expression value of the reference gene (β-actin). Data are presented as mean ± SD from two independent experiments measured at least two times each.
(TIF)

Figure S8 **Contribution of VW-MPSCs to new vessel formation** ***in vivo*** **.** (A, B) TAGLN (red) and hCD34 (green) double-staining shows flattened cells strongly positive for TAGLN (arrowheads) surrounding tightly the vessels formed by HUVEC while also some single roundly shaped and TAGLN-negative cells (arrow) are present probably still representing differentiating MPSCs. Within the plugs the formation of functional new blood vessels was demonstrated by phase contrast microscopy and the presence of erythrocytes within the vessel lumen (B4, C2). (C–F) EGFP-labeling of VW-MPSCs was used in order to follow directly their differentiation into pericytes/SMC and their integration into the wall of new vessels. EGFP labelled VW-MPSCs and HUVEC were grafted as spheroids in Matrigel supplemented with VEGF and FGF2 into Scid mice subcutaneously for 14 days. On sections of removed plug tissue immunofluorescent studies and confocal microscopic analyses were performed. Staining for pericytes/smooth muscle cell makers (C TAGLN; D αSMA; E CD146; F RGS5) and co-localisation of EGFP-fluorescence identified the EGFP-labeled VW-MPSCs as the source of the pericytes and SMC-like cells surrounding the vessels (blue, TOTO®-3 iodide). Bar A, C 20 µm; B, D–F 10 µm.
(TIF)

Figure S9 **Pericyte/smooth muscle cell differentiation of VW-MPSC.** VW-MPSCs were grafted with HUVEC (A, C) as spheroids or alone (B) in Matrigel supplemented with VEGF and FGF2 (A) or TGFß (B) or indicated growth factors (C) into Scid mice subcutaneously for 14 days. On sections of removed plug tissue immunostainings using the TAGLN antibody (brown, DAB staining; hCD34 red, ALP) were performed. TAGLN-positive cells were quantified by counting four randomly chosen optical fields using light microscopy. When VW-MPSCs were grafted together with HUVEC 70%±16 (TGFß; n = 4) and 71%±19 (VEGF/FGF2; n = 2) of all the cells within the plug differentiated into pericytes/smooth muscle cells, and 63%±16 were differentiated when MPSCs were grafted alone (C6). Magnification ×20.
(TIF)

Table S1 **Oligonucleotides used for QRT-PCR.** Specific primers were synthesized based on available sequences for each listened gene. Primer design was done with the program Primer 3 (http://frodo.wi.mit.edu/cgi-bin/primer3/primer3_www.cgi). Cross-reaction of primers with the genes was excluded by comparison of the sequence of interest with a database (Blast 2.2, U.S. National Centre for Biotechnology Information, Bethesda, MD, USA) and all primers used in our study were intron-spanning. PCR products are 300–400 bp in size.
(DOC)
