# Supplementary figures and images for: Vascular Wall-Resident CD44+ Multipotent Stem Cells Give Rise to Pericytes and Smooth Muscle Cells and Contribute to New Vessel Maturation
Source: PLoS One. 2011 May 26;6(5):e20540. doi: 10.1371/journal.pone.0020540 (PMC3102739; doi:10.1371/journal.pone.0020540)

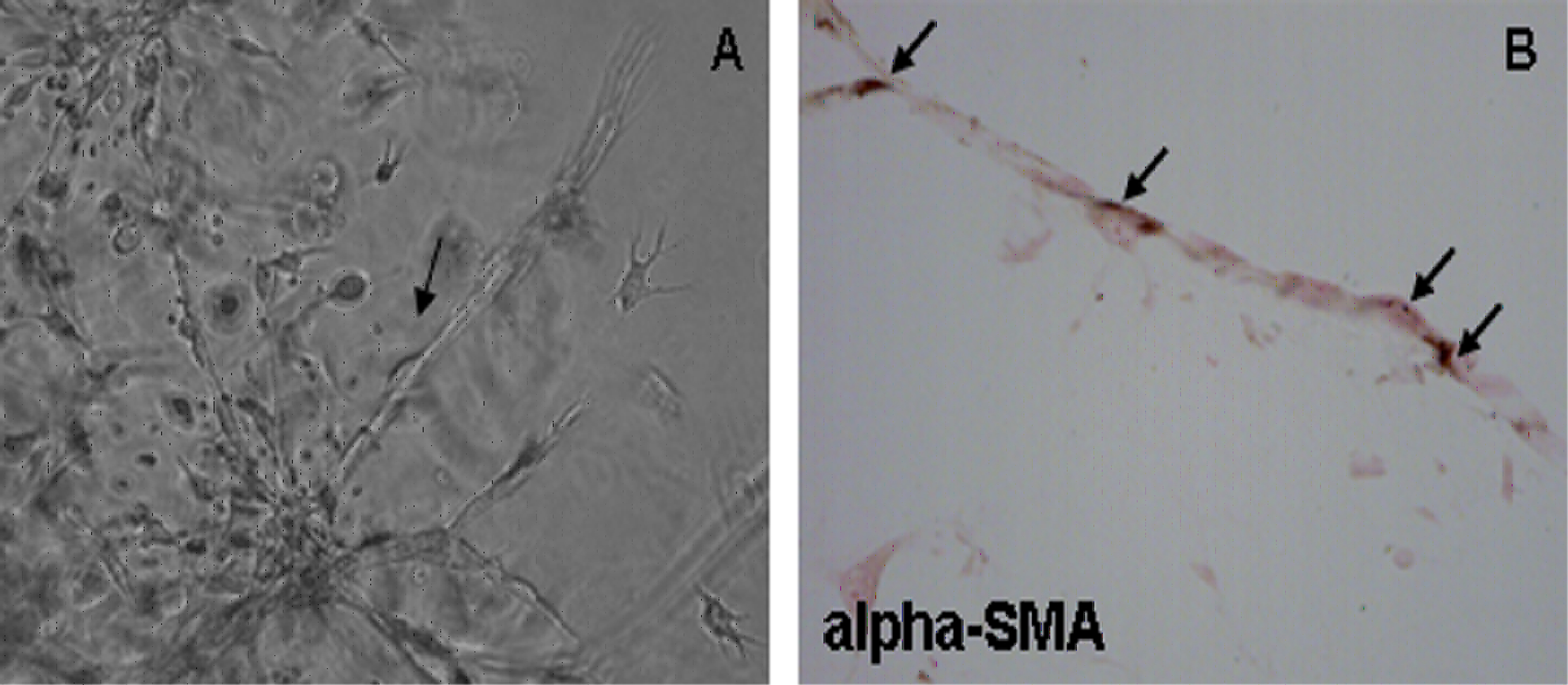

Supplement: Figure S1 — Arterial sprouting assay in Matrigel using fragments of hITA. The capillary-like sprouts from aortic wall demonstrate cellular components (arrows) tightly associated to the vessel sprout from the outside (A). Immunohistochemistry for αSMA on sections from such sprouting tissue shows αSMA-positive (arrows) cells covering the capillary-like structure (red staining) visualized by counterstaining via Calcium red (B). (TIF) [file pone.0020540.s001.tif]

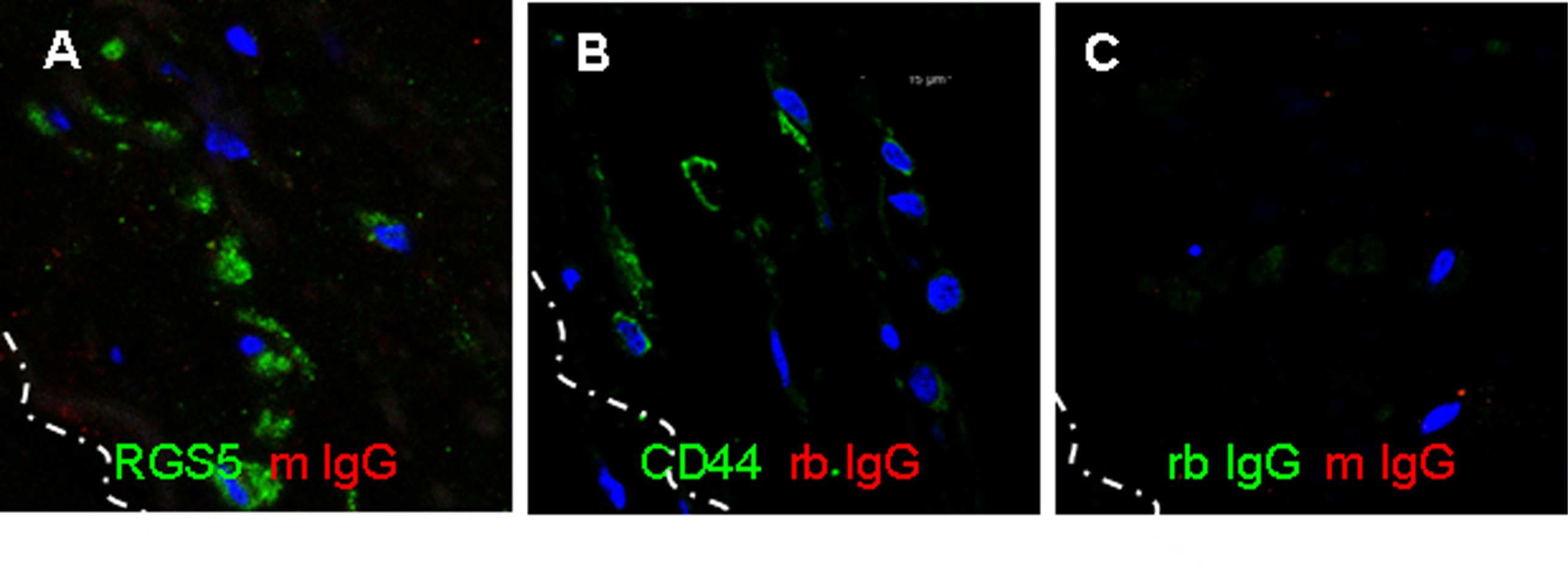

Supplement: Figure S2 — Control stainings of hITA sections for immunofluorescent analysis. In immunofluorescent analysis for CD44+ cells in their native niche we performed double immunostainings on hITA sections combining antibodies against RGS5 (rabbit IgG) and mouse isotype control (A), against CD44 (mouse IgG) and rabbit isotype control (B), as well as mouse and rabbit isotype controls (blue, TOTO®-3 iodide). Dotted line marks the border between media and adventitia of the hITA wall. (TIF) [file pone.0020540.s002.tif]

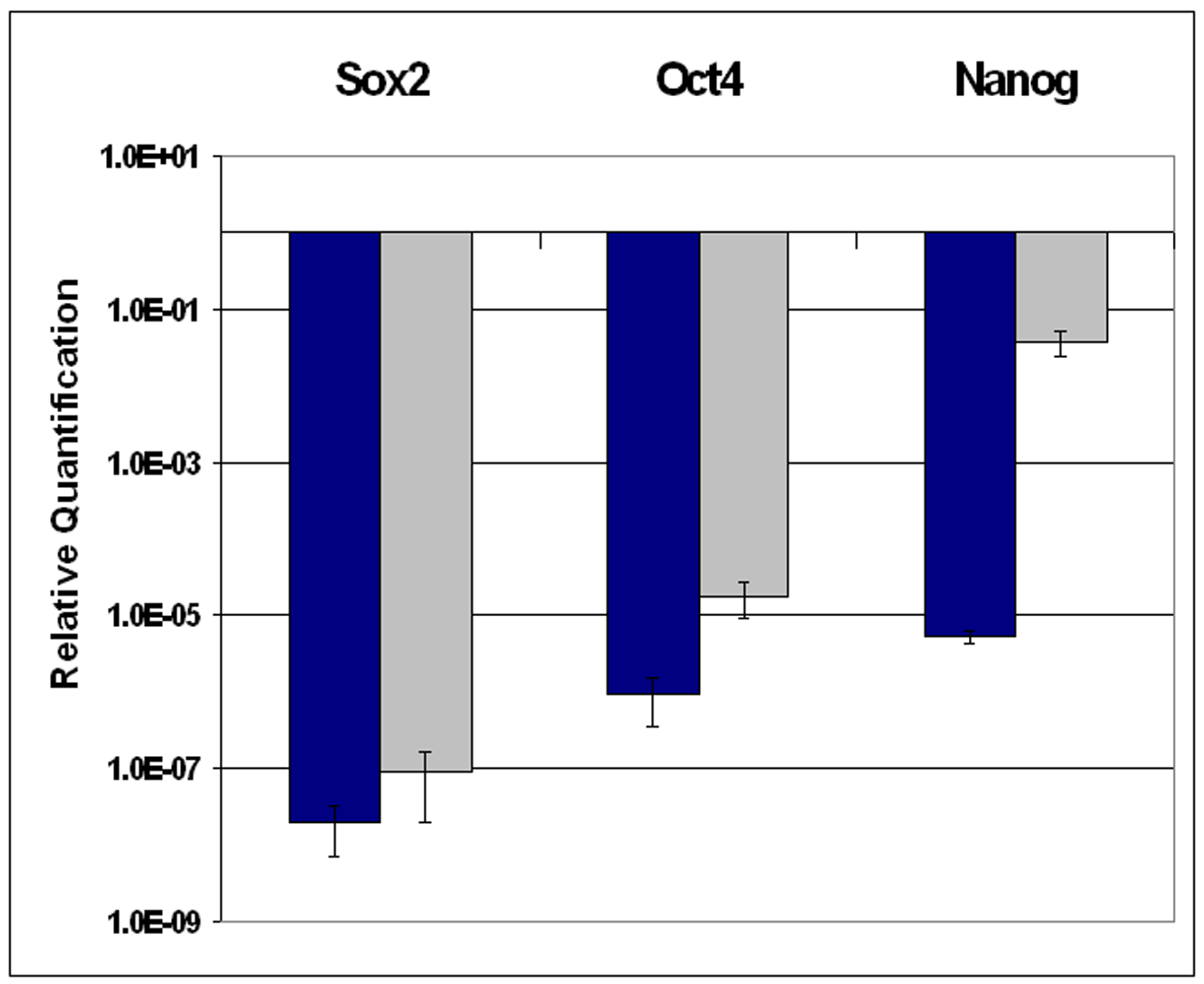

Supplement: Figure S3 — Expression of pluripotent stem cell marker genes in vascular wall-derived MPSCs vs. embryonic stem (ES) cells. QRT-PCR analyses show that genes specific for pluripotent ES (blue bars) cells are partially expressed in VW-MPSCs (grey bars) (Sox2 and to a lower extent Oct4). Y-axis is presented in logarithmic scale. Data are presented as mean ± SD from three independent experiments measured at least two times each. (TIF) [file pone.0020540.s003.tif]

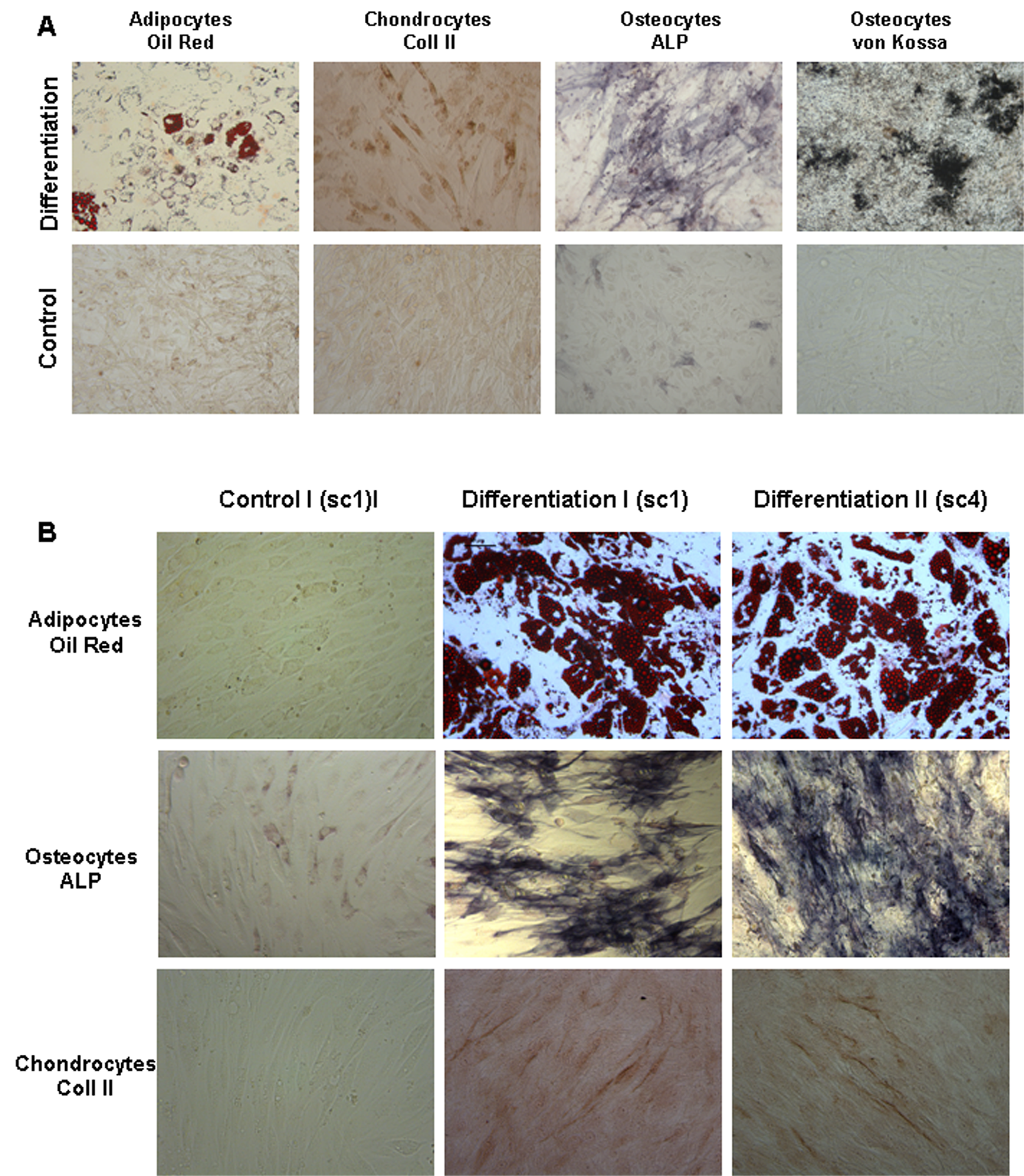

Supplement: Figure S4 — In vitro differentiation of cultured vascular wall-derived CD44+ MPSC. (A) Cultivated vascular wall-derived CD44+ MPSCs differentiate into adipocytes (Oil red), chondrocytes Coll II) and into osteocytes (ALP) within 14 days after induction of differentiation as shown by Oil red staining, by immunostaining for collagen type II (Coll II) and by histochemical staining for alkaline phosphatase (ALP) as well as the von Kossa staining to visualise mineralised calcium (upper figure panel). No specific staining is seen in the corresponding controls (lower figure panel). Magnification ×20. (B) Cultured CD44+ MPSC were clonally expanded by plating primary cell isolates in 96-well cell culture plastic dishes (1 cell per well) as previously described by Chen et al. [R1]. Developed clones were subsequently subjected to obtain a single cell suspension by accutase treatment and re-plated in 96-well plastic dishes (1 cell per well) in order to generate subclones (sc). Different sub-clones (differentiation I, II) differentiate into adipocytes (Oil red staining), into osteocytes (ALP staining) and into chondrocytes (Coll II staining) within 14 days after induction of differentiation. Magnification ×20. [R1] Chen FG, Zhang WJ, Bi D, Liu W, Wei X, Chen FF, Zhu L, Cui L, Cao Y. Clonal analysis of nestin(−) vimentin(+) multipotent fibroblasts isolated from human dermis. J Cell Sci. 2007 Aug 15;120(Pt 16):2875–83. (TIF) [file pone.0020540.s004.tif]

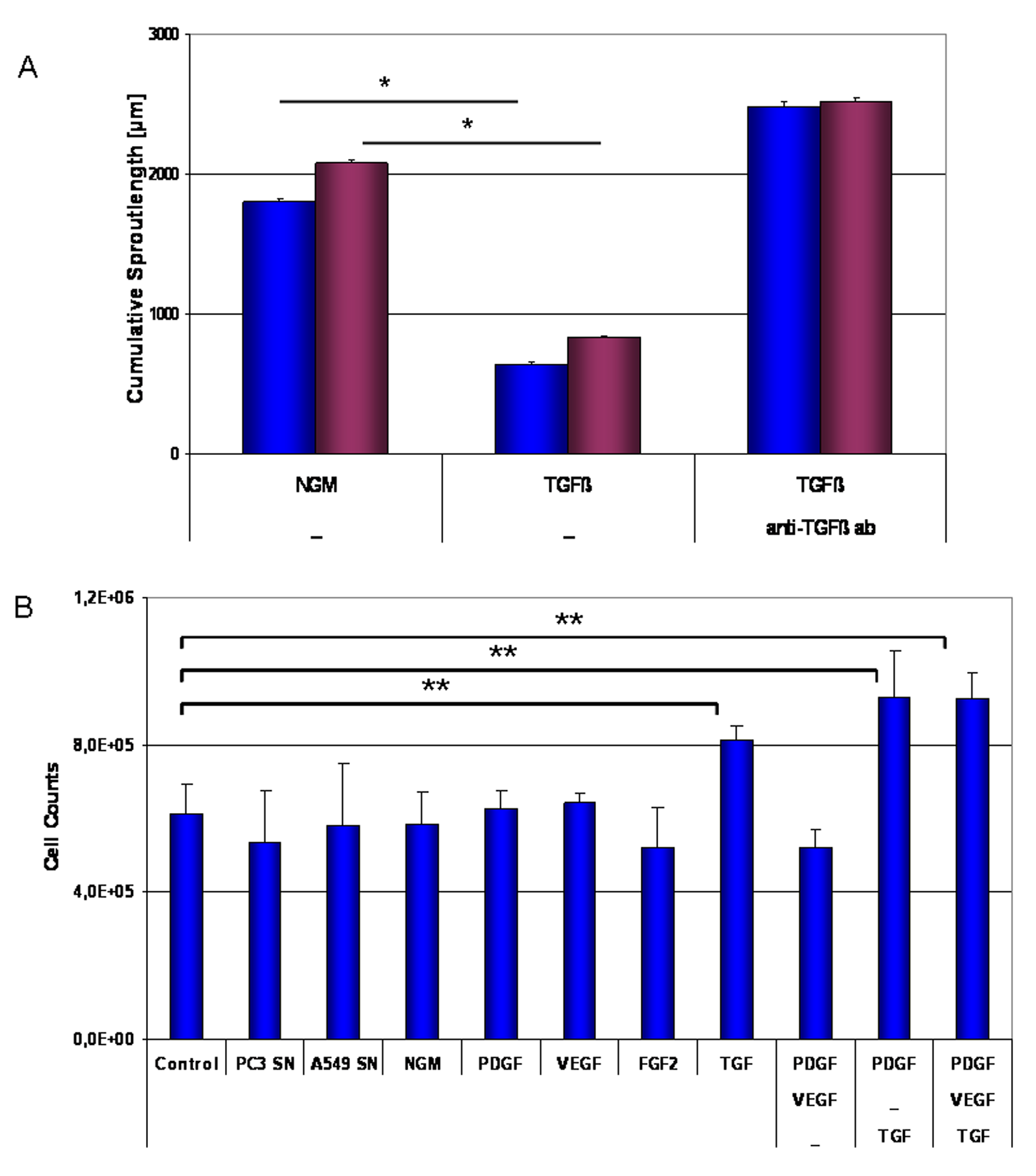

Supplement: Figure S5 — Exogenous TGFβ1 reduces in-gel sprouting and increases cell number of cultured vascular wall-derived MPSCs. (A) VW-derived MPSCs alone (blue bars) or together with endothelial cells (purple bars; ratio 1∶1) were embedded in GFR-Matrigel as 3D-spheroids and exposed to NGM, TGFβ1 (5 ng/ml) or TGFβ1 and TGFβ1 neutralizing antibody (2 µg/ml). In-gel sprouting was quantified after 48 hours of stimulation. The data represent the mean cumulative length of all cord-like sprouts growing from 10 individual spheroids per experimental group. The figure shows the results of 1 of 2 independent experiments with similar results. (B) VW-MPSCs cultured in NGM supplemented with VEGF165, PDGF-BB, FGF2 (10 ng/ml) and TGFβ1 (5 ng/ml) as well as in control and conditioned media of tumor cell lines A549 (A549-SN) or PC3 (PC3-SN) for 14 days show increased cell numbers in response to TGFß alone or in the indicated combinations. Data are presented as mean ± SD from 3 independent experiments performed in duplicates each. ** p≤0.005. (TIF) [file pone.0020540.s005.tif]

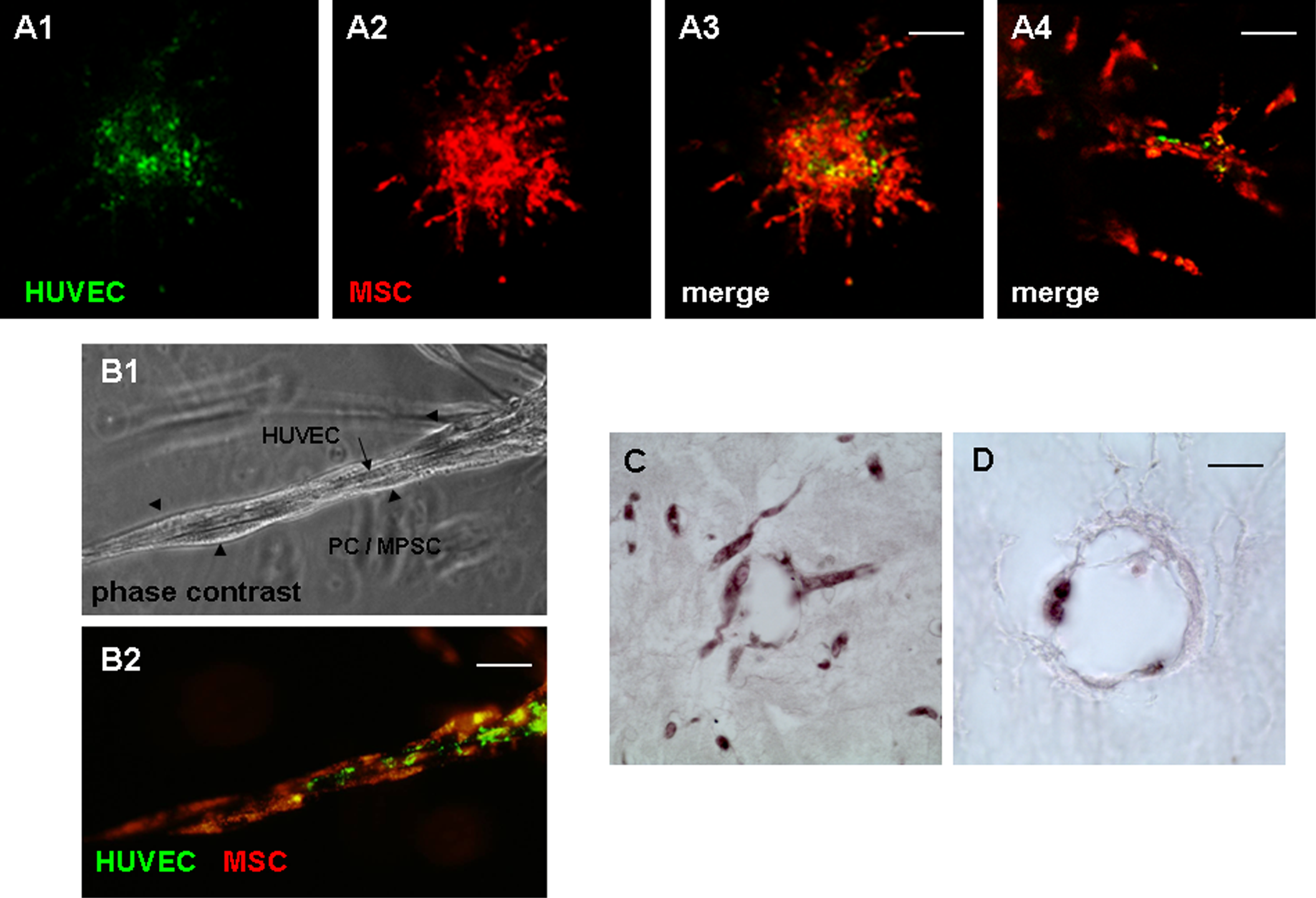

Supplement: Figure S6 — Pericyte-like coverage of endothelial tubes by vascular wall-derived MPSCs in an angiogenic sprouting assay. Pre-labelled VW-MPSCs (green) were seeded together with pre-labelled HUVEC as spheroids (red; ratio 1∶1) in GFR-Matrigel. Capillary-like tube formation was observed within MPSCs/HUVEC after 48 h of culturing. Confocal microscopic analysis was done (A, B1). Bar A3 100 µm, A4 20 µm. Vascular wall-derived MPSCs tightly associate to the tubes formed by HUVEC and wrap them in a pericyte-like manner after prolonged co-culturing (B). Bar 10 µm. Cross-sectioning of samples was followed by a HE-staining in order to demonstrate lumen formation within the capillary tube formation (C, D). Bar10 µm. (TIF) [file pone.0020540.s006.tif]

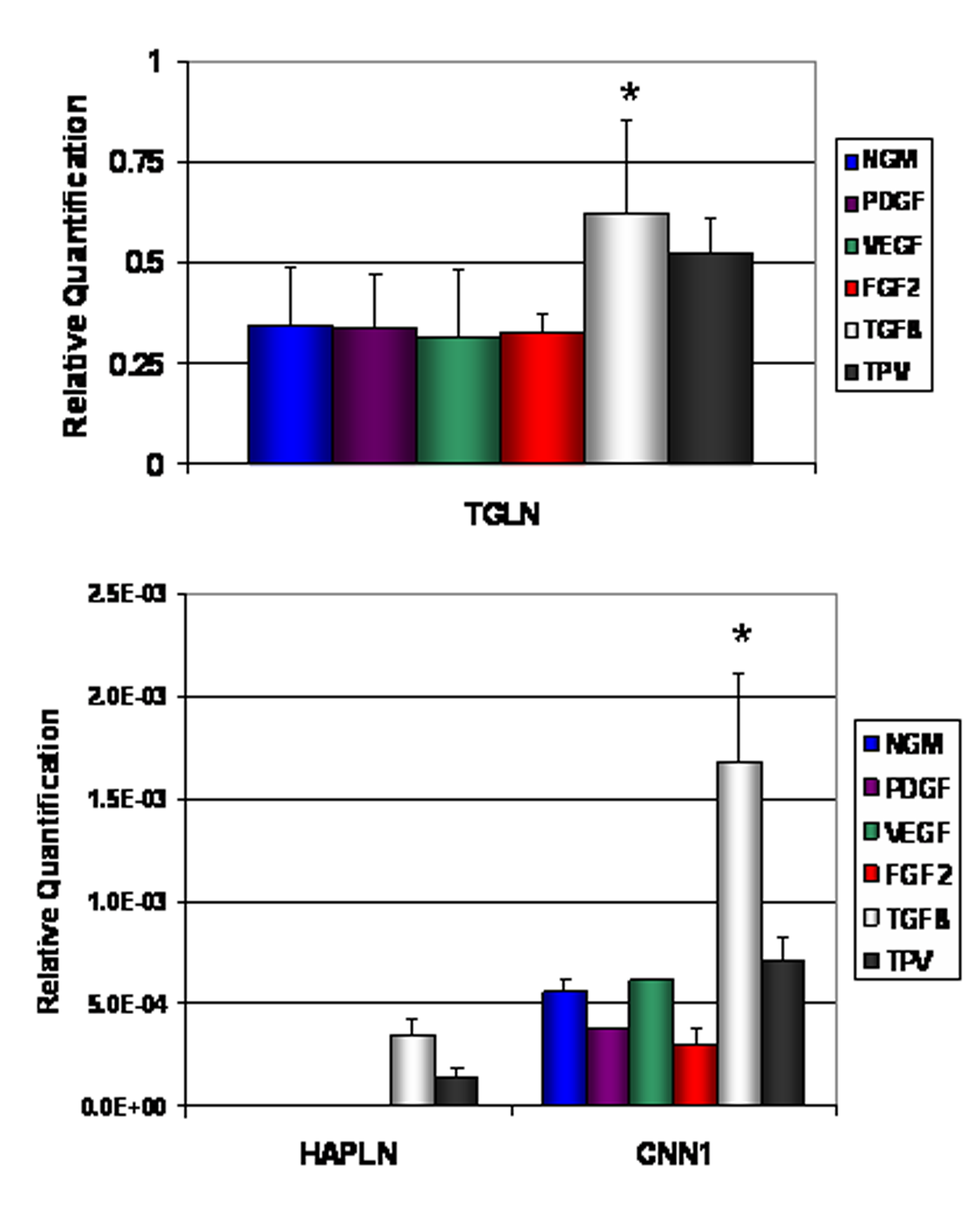

Supplement: Figure S7 — QRT-PCR analyses of vascular wall-derived MPSCs differentiation into SMC upon co-culturing with HUVEC. VW-MPSCs were cultured together with HUVEC (ratio 1∶1) in normal growth media (NGM) or supplemented with VEGF165, PDGF-BB, FGF2 (10 ng/ml), TGFβ1 (5 ng/ml) alone or in indicated combinations. After 14 days cells were trypsinised and subjected for MACS sorting. HUVEC cells were depleted via immunomagnetic beads using CD34 and CD31 antibodies. Total RNA was harvested from VW-MPSCs extracts and subjected for QRT-PCR analysis of TAGLN, HAPLN and CNN1 expression. Resulting expression levels were normalized by division through the mean expression value of the reference gene (β-actin). Data are presented as mean ± SD from two independent experiments measured at least two times each. (TIF) [file pone.0020540.s007.tif]

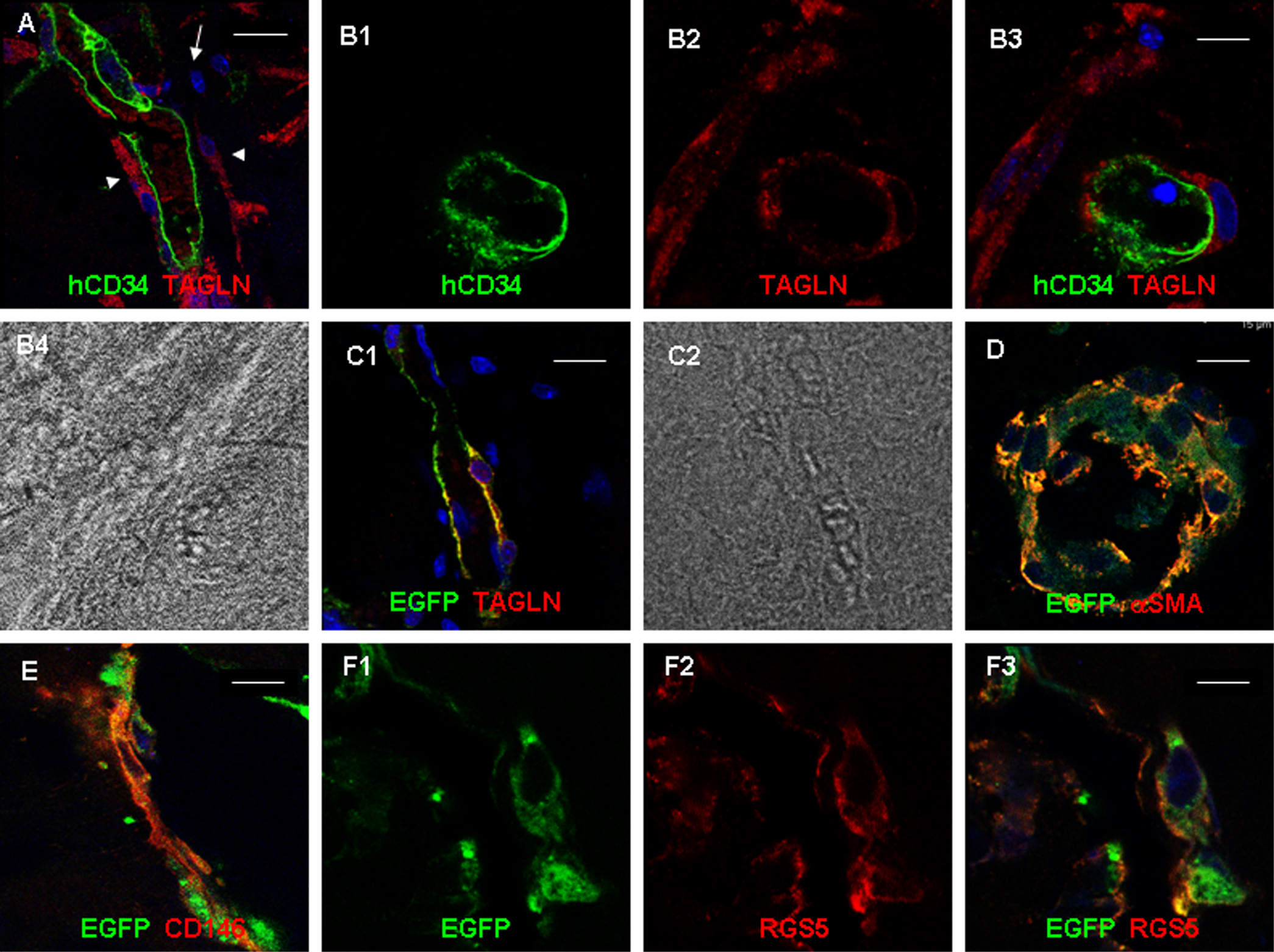

Supplement: Figure S8 — Contribution of VW-MPSCs to new vessel formation in vivo . (A, B) TAGLN (red) and hCD34 (green) double-staining shows flattened cells strongly positive for TAGLN (arrowheads) surrounding tightly the vessels formed by HUVEC while also some single roundly shaped and TAGLN-negative cells (arrow) are present probably still representing differentiating MPSCs. Within the plugs the formation of functional new blood vessels was demonstrated by phase contrast microscopy and the presence of erythrocytes within the vessel lumen (B4, C2). (C–F) EGFP-labeling of VW-MPSCs was used in order to follow directly their differentiation into pericytes/SMC and their integration into the wall of new vessels. EGFP labelled VW-MPSCs and HUVEC were grafted as spheroids in Matrigel supplemented with VEGF and FGF2 into Scid mice subcutaneously for 14 days. On sections of removed plug tissue immunofluorescent studies and confocal microscopic analyses were performed. Staining for pericytes/smooth muscle cell makers (C TAGLN; D αSMA; E CD146; F RGS5) and co-localisation of EGFP-fluorescence identified the EGFP-labeled VW-MPSCs as the source of the pericytes and SMC-like cells surrounding the vessels (blue, TOTO®-3 iodide). Bar A, C 20 µm; B, D–F 10 µm. (TIF) [file pone.0020540.s008.tif]

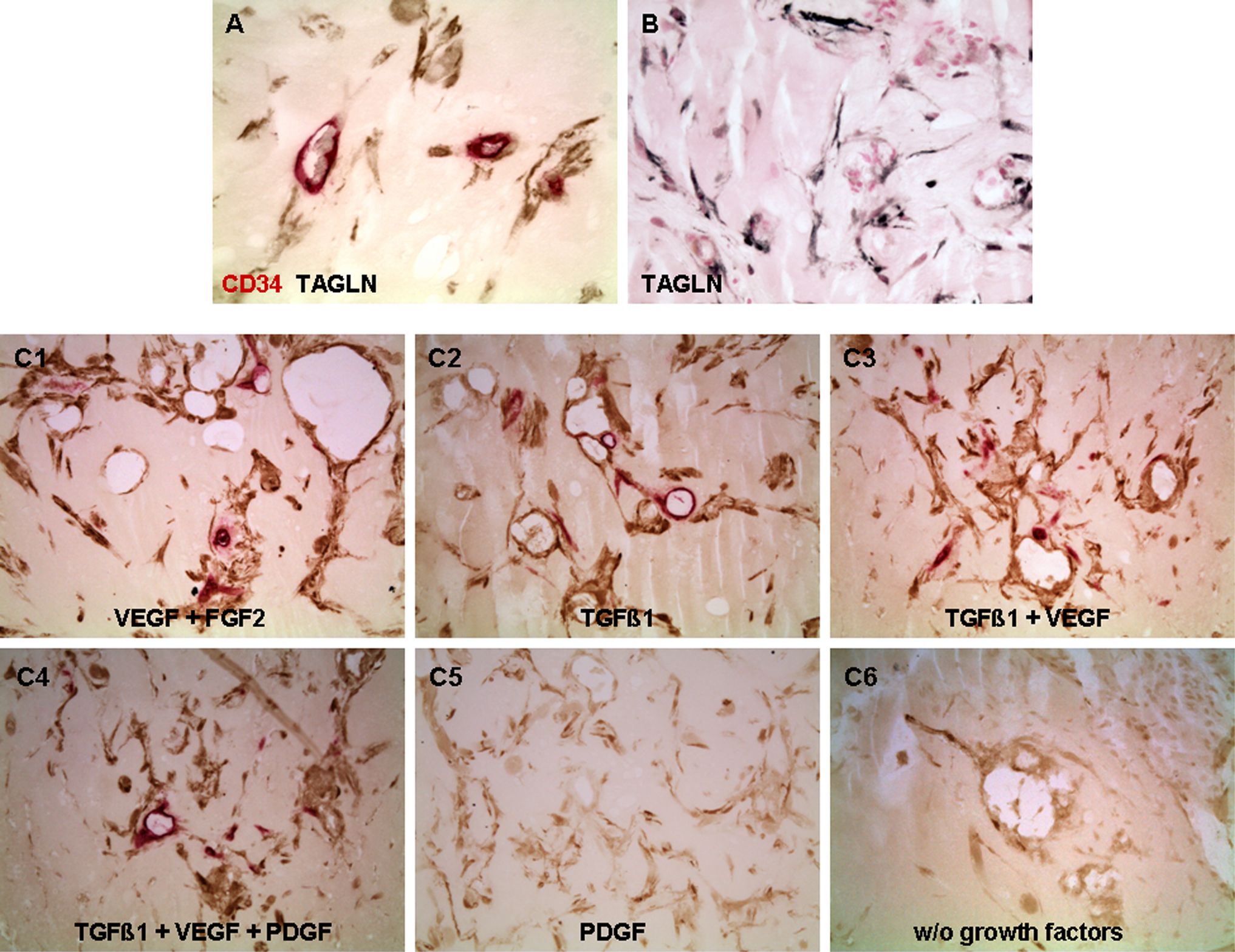

Supplement: Figure S9 — Pericyte/smooth muscle cell differentiation of VW-MPSC. VW-MPSCs were grafted with HUVEC (A, C) as spheroids or alone (B) in Matrigel supplemented with VEGF and FGF2 (A) or TGFß (B) or indicated growth factors (C) into Scid mice subcutaneously for 14 days. On sections of removed plug tissue immunostainings using the TAGLN antibody (brown, DAB staining; hCD34 red, ALP) were performed. TAGLN-positive cells were quantified by counting four randomly chosen optical fields using light microscopy. When VW-MPSCs were grafted together with HUVEC 70%±16 (TGFß; n = 4) and 71%±19 (VEGF/FGF2; n = 2) of all the cells within the plug differentiated into pericytes/smooth muscle cells, and 63%±16 were differentiated when MPSCs were grafted alone (C6). Magnification ×20. (TIF) [file pone.0020540.s009.tif]
