# Supplementary material for: Vascular Wall-Resident CD44+ Multipotent Stem Cells Give Rise to Pericytes and Smooth Muscle Cells and Contribute to New Vessel Maturation
Source: PLoS One. 2011 May 26;6(5):e20540. doi: 10.1371/journal.pone.0020540 (PMC3102739; doi:10.1371/journal.pone.0020540)
Supplement: Table S1 — Oligonucleotides used for QRT-PCR. Specific primers were synthesized based on available sequences for each listened gene. Primer design was done with the program Primer 3 (http://frodo.wi.mit.edu/cgi-bin/primer3/primer3_www.cgi). Cross-reaction of primers with the genes was excluded by comparison of the sequence of interest with a database (Blast 2.2, U.S. National Centre for Biotechnology Information, Bethesda, MD, USA) and all primers used in our study were intron-spanning. PCR products are 300–400 bp in size. (DOC) [file pone.0020540.s010.doc]

|  | | | |
| --- | --- | --- | --- |
| **Gene** | **Primer Sequence** | **Gene** | **Primer Sequence** |
| CD90 fw | ggactgagatcccagaacca | PDGFR-alpha fw | tatgtgccagacccagatgtag |
| CD90 bw | acgaaggctctggtccacta | PDGFR-alpha bw | ctccagggtaagtccattgaag |
| CD73 fw | taaaaggttccaccctgaagaa | PDGFR-beta bw | cagtaaggaggacttcctggag |
| CD73 bw | cttcctgtggaaaacttgatcc | PDGFR-beta fw | cctgagagatctgtggttccag |
| CD105 fw | actcctcccaaggacacttgta | MYOC fw | aggtaacacagcctccatccta |
| CD105 bw | tgatgagctcgacaggatattg | MYOC bw | ccatccattatcccatcaaagt |
| alpha-SMA fw | tggctattccttcgttacta | CNN1 fw | gacgaaaggaaacaaggtgaac |
| alpha-SMA bw | cgatccagacagagtatttgc | CNN1 bw | tcagacagtacttggggtcgta |
| HAPLN fw | tcacagacctcactctggaaga | Desmin fw | cagctactctagctcgcattga |
| HAPLN bw | atattgcacagagccatcactg | Desmin bw | tgacaccttcgacttgtaccac |
| THSP1 fw | gaggggtacagaaacgtagtcg | MYH11 fw | gatttgaagatcaccgatgtca |
| THSP bw | gcacttctttgcactcatcaac | MYH11 bw | ttccagctccttaagctcattc |
| TAGLN fw | tccagactgttgacctctttga | CD146 fw | aaggcaacctcagccatgtcg |
| TAGLN bw | cctctccgctctaactgatgat | CD146 bw | ctcgactccacagtctgggac |
| NG2 fw | gctttgaccctgactatgttggc | beta Actin fw | ggcaccacactttctacaatga |
| NG2 bw | tccagagtagagctgcagca | beta Actin bw | tctctttaatgtcacgcacgat |
|  | | | |
